# Human Cytomegalovirus pUL11, a CD45 Ligand, Disrupts CD4 T Cell Control of Viral Spread in Epithelial Cells

**DOI:** 10.1128/mbio.02946-22

**Published:** 2022-11-29

**Authors:** Samuel A. Osanyinlusi, Jasmin Zischke, Roland Jacobs, Eva M. Weissinger, Thomas F. Schulz, Penelope C. Kay-Fedorov

**Affiliations:** a Institute of Virology, Hannover Medical School, Hannover, Germany; b German Center for Infection Research (DZIF, TTU-IICH), Hannover-Braunschweig Site, Hannover, Germany; c Department of Rheumatology and Clinical Immunology, Hannover Medical School, Hannover, Germany; d Department of Hematology, Hemostasis, Oncology and Stem Cell Transplantation, Hannover Medical School, Hannover, Germany; e Cluster of Excellence 2155 RESIST, Hannover, Germany; University of Kentucky

**Keywords:** CD45, HCMV, IL-10, T cells, cytomegalovirus

## Abstract

Human cytomegalovirus (HCMV) encodes numerous immunomodulatory genes that facilitate its persistence. Previously described mechanisms by which HCMV avoids T cell control typically involve evasion of detection by infected cells. Here, we show that the virus also inhibits T cells directly via an interaction between the pUL11 glycoprotein on infected cells and the CD45 phosphatase on T cells. The antiviral functions of CD4 T cells are impaired as a result of this interaction, largely via induced interleukin 10 (IL-10) secretion in the CD4 T cell central memory compartment, resulting in enhanced viral spread. This establishes CD45 as an inhibitory receptor that regulates antiviral T cell functions and has parallels with the manipulation of natural killer (NK) cells by HCMV. By coculturing donor T cells with HCMV-infected epithelial cells, we observed that CD4 T cells can respond to epithelial cell antigen presentation and can control HCMV spread via cytolytic and cytokine-dependent mechanisms. pUL11 impairs both mechanisms. We showed that pUL11-induced IL-10 secretion requires IL-2, mTOR, and T cell receptor signaling. This characterization of the effects of the pUL11-CD45 interaction may allow for the development of new antiviral therapies and treatments for inflammatory disorders.

## INTRODUCTION

The human cytomegalovirus (HCMV) is a beta herpesvirus with a large double-stranded DNA genome of about 236 kb (1). Depending largely on socio-economic status, HCMV seroprevalence worldwide ranges from 55 to 100% (2). Infection with HCMV is rarely problematic in healthy individuals; however, active HCMV infections in people with weak or compromised immune systems, such as HIV/AIDS patients, transplant recipients, and congenitally infected infants, can produce severe disease. Active HCMV infections can lead to transplant failure or rejection, and about 15% of congenital infections in babies result in permanent sequelae. These negative outcomes can result directly from an HCMV infection, but HCMV also induces a transient immunosuppression that can facilitate secondary bacterial and fungal infections (3, 4).

As with all herpesviruses, once HCMV has established an infection in the host, it will remain lifelong in a latent state with periods of reactivation. In individuals with a weakened immune system, these periods of active infection can result in ineffectively controlled replication of the virus, leading to a high virus load and cytomegalovirus disease (5). Active HCMV infections are controlled by effector T cells with support from other immune cells. Effective control of the virus requires both CD8 and CD4 T cells, with the CD4 T cells exerting both helper and effector functions (6, 7). Effector CD4 cells respond to antigens presented via MHC class II and have been shown to control the spread of HCMV in antigen-presenting cells (APC) that constitutively express MHC class II molecules via cytotoxic and cytokine mediated mechanisms *in vitro* (8). However, other cell types, such as epithelial and endothelial cells, can also upregulate MHC class II expression upon infection or in response to other sources of inflammatory stimuli and may be targets for CD4 T cell killing (9). Epithelial cell infection by HCMV is important, as they are the first cells to be infected following oral transmission and can be infected in large numbers in the lung, GI tract, kidney, and retina (10, 11).

HCMV is capable of complex and multifaceted modulation of its host’s immune responses. Infected cells are manipulated to escape detection. Uninfected immune cells are also affected, resulting in reduced antiviral responses (12). These effects have been studied in detail in NK cells, but T cells are also modulated by the presence of the virus. The mechanisms involved have not yet been fully characterized, but direct contact with infected cells and exposure to the secretome of infected cells contribute. The observed effects on T cells include a reduction in proliferation capacity, regulatory T cell induction, and alterations to their cytokine secretion profiles (13–15).

Interleukin-10 (IL-10) is an immunosuppressive cytokine induced during HCMV infection. IL-10 can limit the functions of APC, thereby impacting their abilities to induce T cell responses. IL-10 also has direct inhibitory effects on many CD4 T cell subtypes, inducing anergy, nonresponsiveness, and a reduction of cytotoxicity (16, 17). IL-10 secretion correlates with a higher viral load during CMV reactivations in sepsis patients, in lower respiratory tract samples from critically ill patients, in HIV positive patients with CMV retinitis, and in solid organ transplant (SOT) recipients (14, 18, 19). Interestingly, the increase of IL-10 in SOT recipients is closely linked to a suppression of CMV-specific T cell responses (20). HCMV infections induce IL-10 from a diverse range of sources, as latently infected and uninfected bystander myeloid progenitor cells secrete IL-10 (21). HCMV-specific CD4 T cells that secrete IL-10 have also been identified in mucosal tissue and in the periphery (8, 22). HCMV additionally encodes viral IL-10 homologs that can enhance cellular IL-10 secretion in DCs (23).

We have shown previously that the HCMV UL11 glycoprotein, a member of the RL11 family of HCMV proteins, can induce IL-10 secretion from CD4 T cells via its interaction with the tyrosine phosphatase CD45 on the plasma membranes of T cells (24, 25). The ligation of CD45 with monoclonal antibodies or nonspecifically via lectins can also lead to IL-10 secretion (26, 27). We have now studied the effects of pUL11 and induced IL-10 on CD4 T cell responses to HCMV. We investigated whether the abilities of CD4 T cells to control HCMV extend beyond dendritic cells to epithelial cells, which are central to the development of initial and recurring HCMV systemic infection. Then, we considered the effects of pUL11 and pUL11-induced IL-10 on HCMV-specific effector functions of CD4 T cells that are driven by epithelial cell infection. We show here that CD4 T cells can control the spread of CMV in infected epithelial cells but that this control is limited by pUL11, largely via IL-10-dependent effects on the T cells themselves. This provides both the first evidence of CD4 T cell control of HCMV infection in epithelial cells and a novel strategy by which HCMV evades it.

## RESULTS

### pUL11 preferentially induces IL-10 secretion in CD4 T cells from HCMV-seropositive donors.

**FIG 1:**
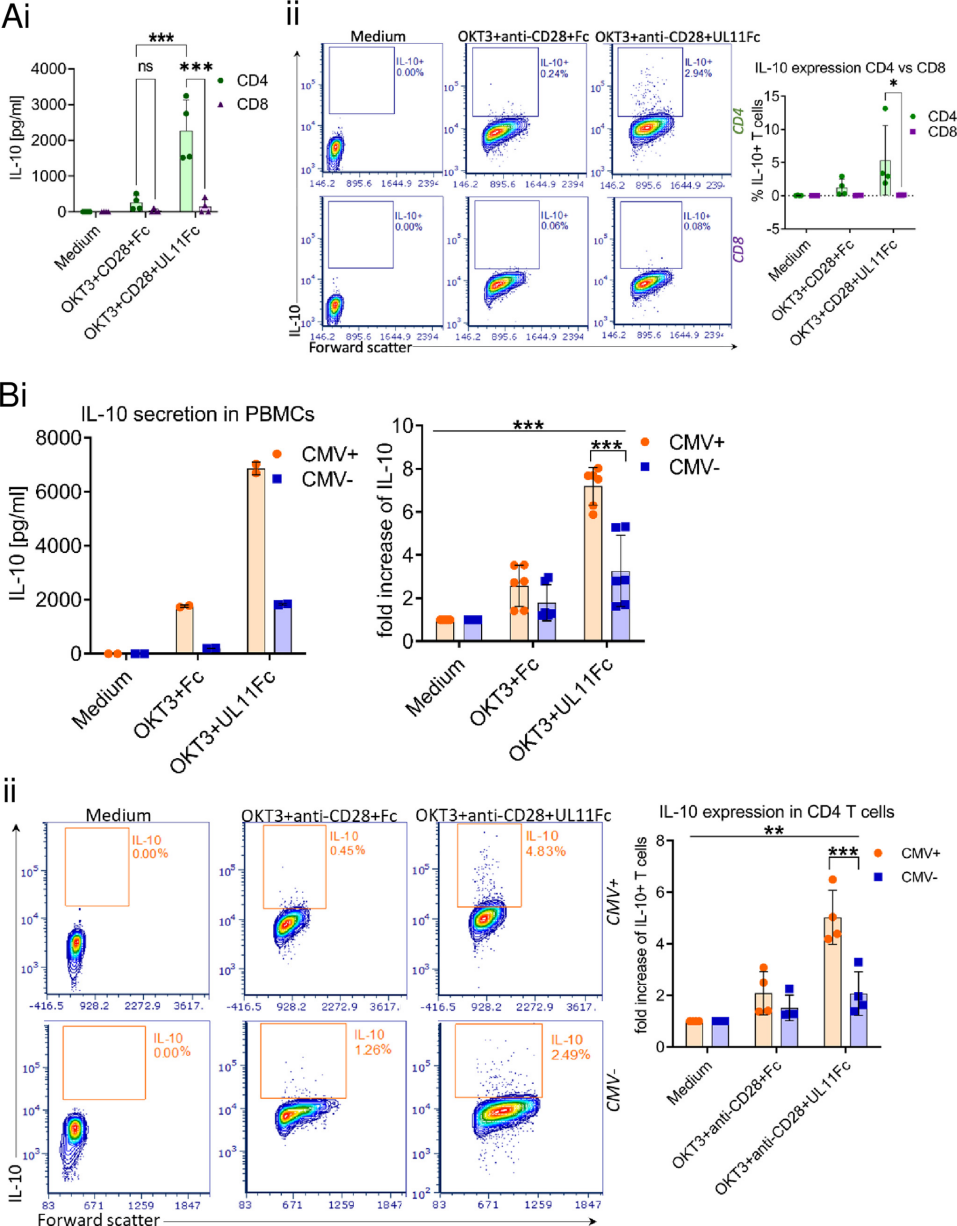
pUL11 preferentially induces IL-10 secretion in CD4 T cells from HCMV–seropositive donors. (A) CD4 or CD8 T cells were untreated (medium) or treated with anti-CD3 (OKT3), anti-CD28, and UL11Fc or Fc for 72 h. Secreted IL-10 was measured via ELISA (i), and IL-10^+^ cells were measured via flow cytometry (ii). T cells were prepared from four donors. (B) PBMC from CMV^+^ and CMV^–^ donors were treated as indicated for 96 h. Secreted IL-10 was measured via ELISA from 6 CMV^+^ and 6 CMV^–^ donors (i), and IL-10^+^ cells were detected via flow cytometry from four CMV^+^ and four CMV^–^ donors. The percentages of live CD3^+^ CD4^+^ IL-10^+^ cells are shown. (ii). The absolute values for one experiment and the fold increases in IL-10 concentration or in the percentage of IL-10^+^ cells over all experiments, normalized to untreated cells, are shown. Error bars represent the standard deviations of biological replicates. Statistical significance was determined via an ANOVA.

pUL11 is a transmembrane glycoprotein that is expressed on the surface of HCMV infected cells. The predicted extracellular domain interacts with the CD45 tyrosine phosphatase and induces the secretion of IL-10 from T cells (24, 25). To determine which type of T cells secretes IL-10 in response to pUL11, we enriched CD4 and CD8 T cells separately, stimulated them via the TCR, and incubated them with the predicted extracellular region of pUL11 fused to the Fc domain of human IgG (UL11Fc) or with the Fc domain alone as a control (Fc) for 72 h. Secreted IL-10 was measured via enzyme-linked immunosorbent assay (ELISA) and intracellular IL-10 was measured via flow cytometry. This showed that the induced IL-10 is largely produced by CD4 T cells (Fig. 1A). Enriching and treating CD4 and CD8 T cells together, rather than separately, resulted in a small increase in the proportion of IL-10-producing CD8 T cells upon TCR stimulation, but it did not alter the fact that the additional pUL11-dependent induction of IL-10 is almost entirely in the CD4 T cell compartment (Fig. S1). We then considered whether this effect was specific to T cells from HCMV seropositive donors. IL-10 is induced in T cells from both HCMV seropositive and seronegative individuals, but the effect is much more pronounced in cells from seropositive donors, in terms of both the amount of IL-10 secreted and the numbers of IL-10 producing cells (Fig. 1B). This result is intriguing, as neither the interaction of pUL11 with CD45 nor the T cell stimulation using anti-CD3 preferentially targets HCMV-specific T cells. Interestingly, not only the total amount of secreted IL-10 but also the number of IL-10 positive CD4 T cells markedly increased following the pUL11 treatment, in comparison to an anti-CD3 only treatment, indicating that not only committed IL-10 producer cells are affected by pUL11. As UL11 is a variable gene, we considered whether pUL11 proteins derived from different strains of HCMV have similar effects. pUL11 from the TB40, Merlin, and Toledo strains of the virus all induce IL-10 secretion preferentially in CD4 T cells from HCMV seropositive donors, achieving approximately similar levels (Fig. S2).

10.1128/mbio.02946-22.1 FIG S1 Induction of IL-10^+^ T cells by pUL11 treatment. Enriched total T cells were untreated (medium) or treated with anti-CD3 (OKT3) and anti-CD28 and UL11Fc or Fc for 72 h. IL-10^+^ cells in the CD4^+^ and CD8^+^ compartments were quantified via flow cytometry. T cells were prepared from five HCMV seropositive donors. Error bars represent the standard deviations of biological replicates. Statistical significance was determined via ANOVA. Download FIG S1, TIF file, 0.9 MB. Copyright © 2022 Osanyinlusi et al. 2022 Osanyinlusi et al. https://creativecommons.org/licenses/by/4.0/ This content is distributed under the terms of the Creative Commons Attribution 4.0 International license.

10.1128/mbio.02946-22.2 FIG S2 Induction of IL-10 secretion by pUL11 from different strains of HCMV. Enriched CD4 T cells were untreated (medium) or treated with anti-CD3 (OKT3), anti-CD28, and 100nM UL11Fc from the TB40, Merlin, or Toledo strains of HCMV or the Fc control for 96 h. Secreted IL-10 was measured via ELISA. Cells were prepared from four HCMV seropositive and two HCMV seronegative donors. Error bars represent the standard deviations of biological replicates. Statistical significance was determined via ANOVA. Download FIG S2, TIF file, 0.4 MB. Copyright © 2022 Osanyinlusi et al. 2022 Osanyinlusi et al. https://creativecommons.org/licenses/by/4.0/ This content is distributed under the terms of the Creative Commons Attribution 4.0 International license.

### pUL11 induces IL-10 secretion largely in the central memory (T_CM_) subset of the CD4 T cells.

**FIG 2:**
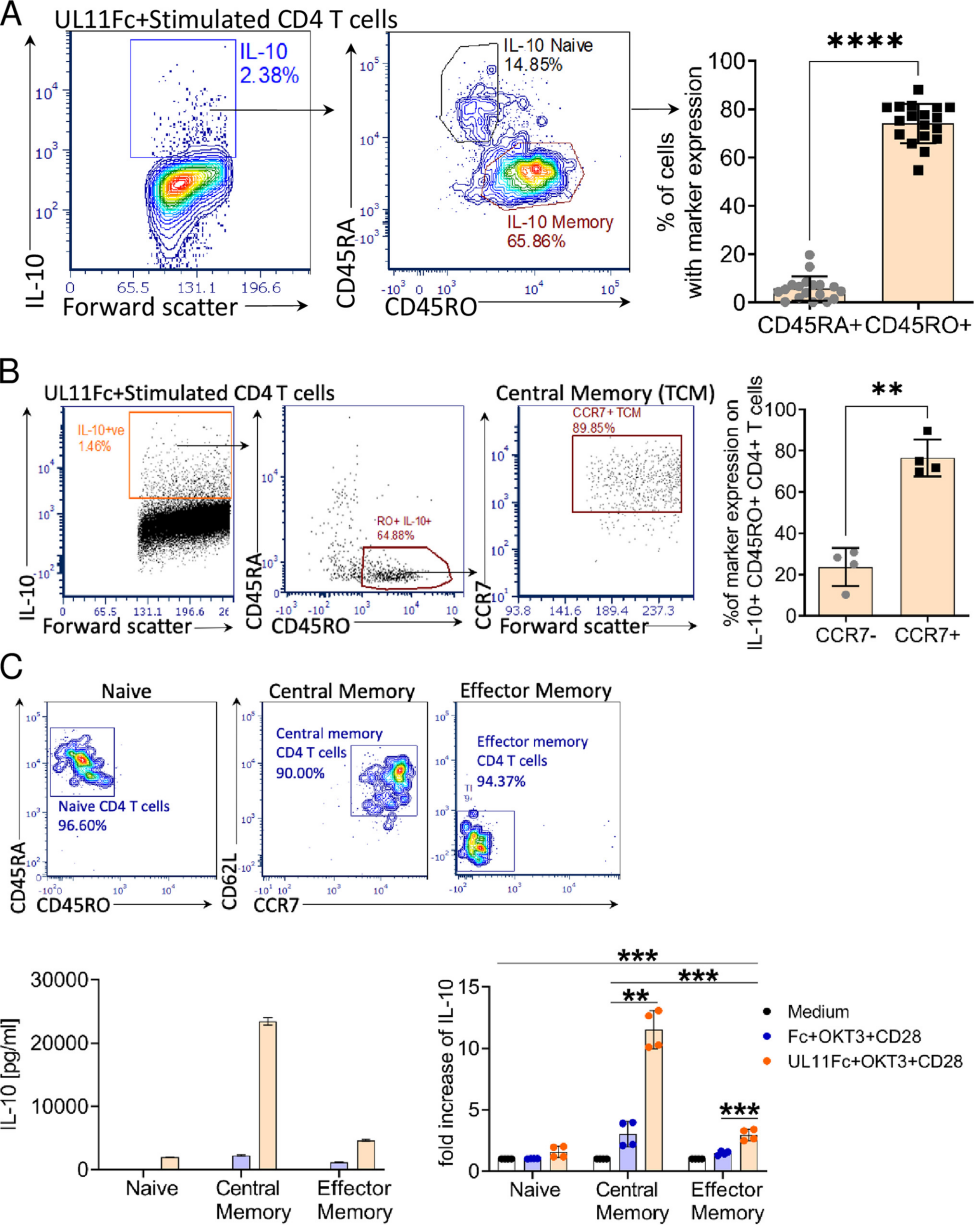
pUL11 induces IL-10 secretion largely in central memory CD4 T cells. (A) CD4 T cells were stimulated with anti-CD3 (OKT3) and anti-CD28, and they were treated with UL11Fc for 72 h. Cells were labeled extracellularly with anti-CD45RA and anti-CD45RO, and they were labeled intracellularly with anti-IL-10 before measurement via flow cytometry. Naive (CD45RA) and memory (CD45R0) live IL-10^+^ cells from one representative experiment as well as percentages from replicates from 18 donors are shown. (B) CD4 T cells were treated as in (A) for 72 h. Cells were labeled extracellularly with anti-CD45RA, anti-CD45RO, and anti-CCR7, and they were labeled intracellularly with anti-IL-10 before measurement via flow cytometry. Live IL-10^+^ cells are shown with the expression of naive and memory markers, CCR7 expression for one representative experiment, and percentages of CCR7 expression in IL-10^+^ CD45R0^+^ CD4 T cells from four donors. (C) CD4 T cells were sorted via FACS into naive (CD45RA^+^) and memory (CD45R0^+^) populations. Memory cells were then sorted into central memory (CD62L^+^ CCR7^+^) and effector memory (CD62L^−^ CCR7^−^) cells. Each fraction was treated with anti-CD3, anti-CD28, and Fc or UL11Fc for 96 h, and secreted IL-10 was measured via ELISA. One representative experiment out of four is shown, using cells from four donors. The fold increases in secreted IL-10 are shown for all four experiments. Error bars represent the standard deviations of the biological replicates. Statistical significance was determined via *t* tests (A and B) and an ANOVA (C).

pUL11 induces IL-10 secretion more effectively in CD4 T cells from CMV seropositive donors than in CD4 T cells from CMV seronegative donors. This difference between seronegative and seropositive donors implies that pUL11 may act via a memory compartment of the CD4 T cells. Therefore, we used surface markers for naive (CD45RA) and memory (CD45R0) T cells to characterize the induced IL-10^+^ cells, which showed that the majority of the IL-10^+^ cells after the pUL11 treatment were from the memory compartment (Fig. 2A). The CD4 memory compartment can be further divided into central memory and effector memory cells by additional surface marker expression. CCR7 and CD62L are responsible for homing to secondary lymphoid organs and are expressed on central memory T cells (28). The great majority of IL-10 secreting T cells following pUL11 treatment expressed CCR7 and were therefore central memory cells (Fig. 2B). As pUL11 treatment could potentially affect T cell maturation markers, we also determined which starting populations of untreated cells can generate IL-10 upon pUL11 treatment. Sorting the CD4 T cells to separate the naive, central memory and effector memory cells by CD45RA, CD45R0, CCR7, and CD62L expression prior to treatment with pUL11 showed that preexisting central memory cells were responsible for most of the pUL11-induced IL-10 secretion (Fig. 2C). Therefore, CD4 central memory T cells maintain their surface marker characteristics during the induction process and form the majority of the pUL11-induced IL-10 secreting cells. The enhanced IL-10 induction in CD4 T cells from HCMV seropositive donors could potentially be due to the presence of more CD4 central memory T cells in the PBMC isolated from these donors than in those isolated from HCMV seronegative individuals. Therefore, we compared the percentages of central memory T cells within CD4 T cells from eight HCMV seropositive donors and eight HCMV seronegative donors with the percentages of IL-10 positive cells following pUL11 treatment. There was high variation between donors, with HCMV seropositive donors frequently having high percentages of central memory T cells, but there was no direct correlation between the percentages of central memory cells and IL-10 positive cells (Table S1).

10.1128/mbio.02946-22.10 TABLE S1 Enriched CD4 T cells were stimulated with OKT3 and anti-CD28 and treated with 100nM UL11Fc for 48 h. The percentages of live IL-10^+^ cells and CD45R0^+^ CCR7^+^ central memory CD4 T (TCM) cells were measured via flow cytometry. Download Table S1, TIF file, 0.1 MB. Copyright © 2022 Osanyinlusi et al. 2022 Osanyinlusi et al. https://creativecommons.org/licenses/by/4.0/ This content is distributed under the terms of the Creative Commons Attribution 4.0 International license.

### pUL11 has an inhibitory effect on CD4 T cell control of HCMV spread.

HCMV infection can be controlled by effector CD4 T cells (8, 29). As pUL11 perturbs CD4 T cell function and induces IL-10, which can directly limit CD4 T cell activation and expansion, we investigated whether the presence of pUL11 can influence CD4 T cell control of an HCMV infection. CD4 T cell control of HCMV spread in APC has been described, and we wanted to understand whether the spread of infections can be similarly restricted in other relevant cell types. Epithelial cell infection contributes to the spread and pathology of HCMV infections in the lung, GI tract, kidney, and retina, making them suitable choices for these experiments (11, 30). We used the retinal pigment epithelial (RPE) cell line and PBMC from HCMV seropositive donors. The Merlin strain of HCMV can infect epithelial cells efficiently via cell to cell spread (31). We made use of a GFP-expressing derivate of the Merlin strain (HCMV Merlin GFP) and a UL11 deletion mutant of the same virus (HCMV Merlin dUL11 GFP) (32). Using GFP expression as an approximate method by which to detect HCMV-infected cells, we could show that both the HCMV Merlin GFP and the HCMV Merlin dUL11 GFP viruses grow equally well in RPE cells (Fig. S3).

10.1128/mbio.02946-22.3 FIG S3 HCMV growth curves in RPE cells. (i) Fluorescent microscopic images of HCMV Merlin GFP (WT) or HCMV Merlin dUL11 GFP (dUL11) infection in RPE cells. (ii) GFP-expressing cells in cultures of RPE cells infected with HCMV Merlin GFP (WT) or HCMV Merlin dUL11 GFP (dUL11) at an MOI of 0.5 were quantified via flow cytometry over 17 days in culture. Download FIG S3, TIF file, 0.6 MB. Copyright © 2022 Osanyinlusi et al. 2022 Osanyinlusi et al. https://creativecommons.org/licenses/by/4.0/ This content is distributed under the terms of the Creative Commons Attribution 4.0 International license.

**FIG 3:**
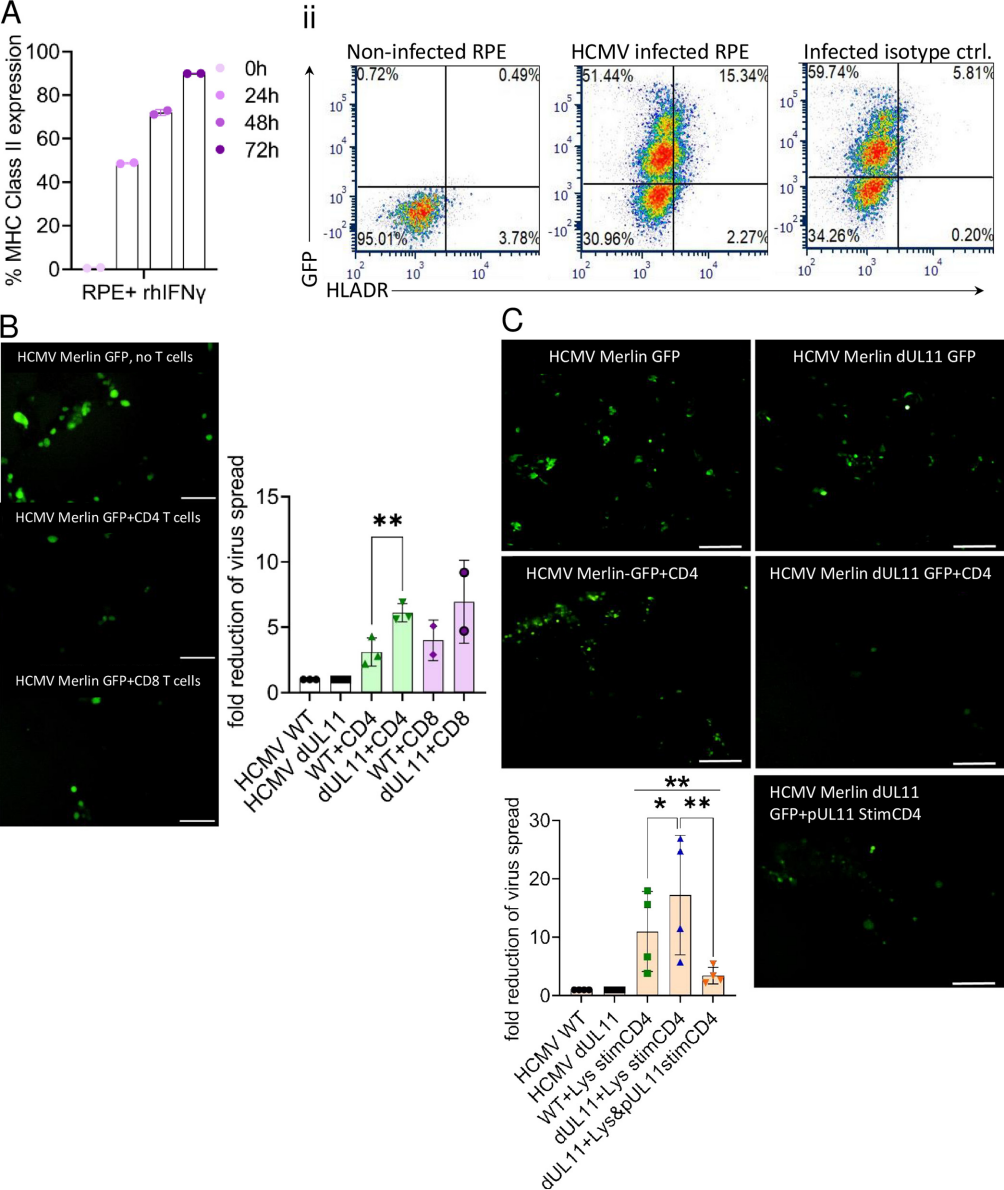
pUL11 has an inhibitory effect on the CD4 T cell control of HCMV spread. (A) Retinal pigment epithelial (RPE) cells were treated with rhIFN-γ for the indicated time points (i), infected with HCMV Merlin GFP (infected), or left uninfected (ii). MHC Class II^+^ cells were measured via flow cytometry. MHC Class II^+^ cells are shown for one representative experiment out of three (i) and four (ii). (B and C) Enriched CD4 or CD8 T cells were stimulated with lysate from HCMV-infected cells and anti-CD28 for 24 h (B), or were additionally stimulated with UL11Fc (C) and cocultured with HCMV Merlin GFP or HCMV Merlin dUL11 GFP-infected RPE cells for 7 days at a ratio of RPE cells:T cells of 1:3. Fluorescent images at a magnification of 10× are shown. Scale bar= 100 μm. The quantitation of GFP^+^ RPE cells, measured via FACS, is shown as the fold reduction of the virus spread, normalized to HCMV Merlin GFP or HCMV Merlin dUL11 GFP-infected RPE cells, as appropriate, without T cells. CD4 T cells from three CMV^+^ donors and CD8 T cells from two CMV^+^ donors were used in (B), and CD4 T cells from 4 CMV^+^ donors were used in (C). Error bars represent the standard deviations of biological replicates. The statistical significance of the differences between groups was determined via one-way ANOVA.

RPE cells exposed to IFN-γ express MHC II on their surface. A minority of HCMV infected RPE cells also express MHC II, despite the existence of several viral gene products that act to disrupt MHC II expression (Fig. 3A). Although epithelial cells expressing MHC II can process and present endogenous antigen to CD4 T cells and can express some costimulatory molecules, they are not as effective in antigen presentation as are canonical antigen-presenting cells, such as dendritic cells (33). Therefore, we used a method in which we loaded antigen-presenting cells with lysate from HCMV-infected cells to provide the initial recall stimulus for HCMV memory CD4 T cells. The infected RPE cells then served as targets of these recall responses. T cells enriched from PBMC contain residual levels of MHC class II expressing antigen-presenting cells (Fig. S4A), which is sufficient for T cell activation (34). These APC-containing T cells were incubated with HCMV cell lysate for 24 h. Then, the lysate was removed, and the activated enriched T cells were added to uninfected or virus-infected RPE cells for 7 days at an effector to target ratio of 3:1. The control of the infection was monitored by quantifying the percentage of GFP-expressing RPE cells via flow cytometry. Using this coculture model, CD4 T cells can control HCMV infection in RPE cells almost as effectively as can CD8 T cells, as measured by a reduction in the number of GFP-expressing cells, measured via flow cytometry (Fig. 3B). Without pretreatment with the HCMV lysate, both CD4 and CD8 T cells showed low levels of control of infected cells, indicating that the RPE cells can present the antigen but cannot provide the initial activation stimulus for T cells (Fig. S4B). We also saw no evidence of alloreactions between the T cells and the RPE cells, presumably also due to the reduced costimulatory functions of RPE cells, as has been previously described (35, 36). We typed the HLA-A, -B, -DR, -DP, and –DQ of RPE cells and donors, and we found no increased cytotoxicity in cocultures with uninfected RPE cells when matching between the HLA molecules on the T cells and the RPE cells was lacking (even in the presence of IFN-γ to upregulate MHC II on the RPE cells) (Fig. S5A and B). Also, there was no increase in the nonspecific loss of HCMV-infected cells upon coculture with T cells from HCMV seronegative mismatched donors (Fig. S6A). Therefore, this new coculture model appears to be useful for investigating CD4 T cell control of HCMV spread in epithelial cells.

10.1128/mbio.02946-22.4 FIG S4 MHC class II expressing antigen-presenting cells in enriched CD4 T cells are functional. (A) CD4 T cells from two HCMV seropositive donors were enriched and stained with anti-HLADR APC and acquired via flow cytometry. One representative experiment is shown. (B) CD4 or CD8 T cells from two HCMV seropositive donors were left untreated (–CMV lysate) or stimulated with lysate from HCMV infected cells and anti-CD28 (+CMV lysate) for 24 h and cocultured with HCMV Merlin GFP-infected RPE cells for 7 days at a ratio of RPE cells:T cells of 1:3. The quantitation of the GFP^+^ RPE cells, as measured via FACS, is shown as the fold reduction of virus spread, normalized to HCMV Merlin GFP-infected RPE cells without T cells. Error bars represent the standard deviations of biological replicates. Download FIG S4, TIF file, 0.4 MB. Copyright © 2022 Osanyinlusi et al. 2022 Osanyinlusi et al. https://creativecommons.org/licenses/by/4.0/ This content is distributed under the terms of the Creative Commons Attribution 4.0 International license.

10.1128/mbio.02946-22.5 FIG S5 Cytotoxic effects are mainly due to T cells from HCMV seropositive donors responding to HCMV infected RPE cells. (A and B) RPE cells that were uninfected (RPE) or infected with HCMV Merlin GFP (WT) were cultured with enriched CD4 or CD8 T cells from HCMV seropositive (CMV^+^) or HCMV seronegative (CMV^–)^ donors that had been pre-stimulated with HCMV-infected cell lysate and anti-CD28 for 24 h in the presence of 5μg IFN-γ, where indicated, for 7 days. (C) Enriched CD4 T cells were untreated or pre-stimulated with HCMV lysate (CD4^+^Lys) for 24 h. Cells were removed from lysate and then incubated for 7 days in the presence of pUL11, where indicated. (A–C) Cytotoxicity was determined via lactate dehydrogenase (LDH) release and is shown as a percentage of LDH release from fully lysed cells as described in Materials and Methods. In panel A, cells from 2 CMV^+^ and 2 CMV^–^ donors were used. In panel B, 2 CMV^+^ donors were used. For panel C, cells from 3 CMV^+^ donors were used. (D) HLA typing of the RPE cells and T cells used in panels A and B. Download FIG S5, TIF file, 0.1 MB. Copyright © 2022 Osanyinlusi et al. 2022 Osanyinlusi et al. https://creativecommons.org/licenses/by/4.0/ This content is distributed under the terms of the Creative Commons Attribution 4.0 International license.

10.1128/mbio.02946-22.6 FIG S6 T cell responses to infected RPE cells are restricted to cells from HCMV seropositive donors. RPE cells that were uninfected (RPE) or infected with HCMV Merlin GFP (WT) or HCMV Merlin dUL11 GFP (dUL11) were cultured with CD4 T cells from HCMV seronegative donors (A) or from HCMV seronegative (CMV^–^) or HCMV seropositive (CMV^+^) donors as indicated (B) that had been pre-stimulated for 24 h with HCMV-infected cell lysate and anti-CD28 for 7 days. (A) The quantitation of GFP^+^ RPE cells, as measured via FACS, is shown as the fold reduction of virus spread, normalized to HCMV Merlin-GFP or HCMV Merlin dUL11 GFP infected RPE cells without T cells as appropriate. The values from three experiments using CD4 T cells from three different CMV^–^ donors and CD8 T cells from one CMV^–^ donor are shown. (B) IL-10 secretion into the supernatant was measured via ELISA. Values for T cells derived from two HCMV seropositive and two HCMV seronegative donors are shown. Error bars represent the standard deviations of biological replicates. (C) HLA typing of RPE cells and T cells used in panels A and B. Download FIG S6, TIF file, 0.1 MB. Copyright © 2022 Osanyinlusi et al. 2022 Osanyinlusi et al. https://creativecommons.org/licenses/by/4.0/ This content is distributed under the terms of the Creative Commons Attribution 4.0 International license.

To determine the effect of pUL11, we infected RPE cells with HCMV Merlin GFP or HCMV Merlin dUL11 GFP and compared the virus spread in the presence of CD4 or CD8 T cells, using the coculture system. A clear increase in the effectiveness of both CD4 and CD8 T cell control could be seen in the absence of pUL11, as indicated by a greater reduction in the virus spread in HCMV Merlin dUL11 GFP-infected cultures than in HCMV Merlin GFP infections in the presence of CD4 or CD8 T cells (Fig. 3B). As the induction of IL-10 by pUL11 was almost exclusively present in CD4 T cell cultures, we continued to focus on CD4 T cell effects in order to understand the importance of this effect. To confirm that the increased reduction in infected cell numbers seen in the HCMV Merlin dUL11 GFP-infected cocultures was due to the loss of pUL11, we added purified pUL11Fc back into the HCMV Merlin dUL11 GFP infected cocultures. The increase in T cell control of infected cells was reversed to that seen in the HCMV Merlin GFP-infected cocultures (Fig. 3C). Therefore, the presence of pUL11 inhibits CD4 T cell control of HCMV infections in epithelial cells.

### IL-10 induction by pUL11 is largely responsible for the reduction in CD4 T cell efficacy.

**FIG 4:**
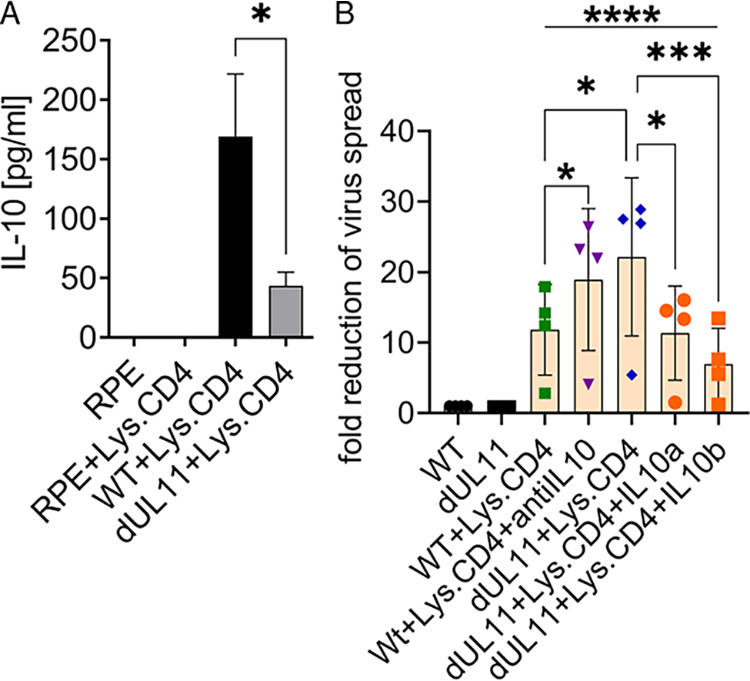
IL-10 induction by pUL11 reduces CD4 T cell efficacy. RPE cells that were uninfected (RPE), infected with HCMV Merlin GFP (WT), or infected with HCMV Merlin dUL11 GFP (dUL11) were cultured alone or with CD4 T cells that had been stimulated with HCMV-infected cell lysate and anti-CD28 (Lys. CD4) for 96 h at a ratio of RPE cells:T cells of 1:3. (A) Secreted IL-10 was measured via ELISA, using T cells from two CMV^+^ donors. (B) An IL-10 blocking antibody (anti-IL-10) or exogenous IL-10 at 125 pg/mL (IL10a) or 500 pg/mL (IL10b) was added to the culture for 7 days. The quantitation of GFP^+^ RPE cells, measured via flow cytometry, is shown as the fold reduction of the virus spread, normalized to HCMV Merlin-GFP or to HCMV Merlin dUL11 GFP-infected RPE cells, as appropriate, without T cells. The experiment was performed four times, using T cells from four CMV^+^ donors. Error bars represent the standard deviations of biological replicates.

We determined whether HCMV infected epithelial cells could induce IL-10 in CD4 T cells in our coculture system. Enriched CD4 T cells from HCMV seropositive donors were pre-stimulated with lysate from HCMV infected cells and cultured with RPE cells. When the RPE cells were infected with the HCMV Merlin GFP parental virus, a robust induction of IL-10 in the coculture supernatant could be detected via ELISA (Fig. 4A). Uninfected RPE cells did not induce any IL-10 secretion. Infection of the RPE cells with HCMV Merlin dUL11 GFP showed markedly reduced IL-10 induction in comparison to that induced by the parental virus. The production of IL-10 was not completely abrogated, indicating that pUL11 is not the only viral inducer of IL-10, as we have previously seen (25). No IL-10 was induced under these conditions using enriched CD4 T cells from HCMV seronegative donors, showing that the effect was specific (Fig. S6B). The ELISA kit used here detects cellular IL-10 but does not detect the HCMV IL-10 homologue cmvIL-10. cmvIL-10 is expressed to similar levels in RPE cells that are infected with both the HCMV Merlin GFP and HCMV Merlin dUL11 GFP viruses, indicating that pUL11 does not affect its expression (Fig. S7). Then, we investigated whether the presence of IL-10 is responsible for the pUL11-dependent effects on CD4 T cell control of the virus. With the addition of an anti-IL-10 blocking antibody (37), CD4 T cell control of HCMV Merlin GFP infection increased almost to the level observed with the HCMV Merlin dUL11 GFP infections (Fig. 4B). This blocking antibody only affects cellular IL-10 and does not bind or neutralize cmvIL-10 (38). The degree of control of HCMV Merlin GFP infections by CD4 T cells in the absence of the anti-IL-10 block indicates T cell capacities that remain functional, despite the presence of induced IL-10 and pUL11. We added rhIL-10 to HCMV Merlin dUL11 GFP-infected cocultures to determine whether the presence of IL-10 is sufficient to inhibit the increased CD4 T cell control of this virus. The addition of comparable amounts of IL-10 to those present in the HCMV Merlin GFP-infected cocultures resulted in decreased CD4 T cell control of the viral spread, equal to and beyond that seen for the HCMV Merlin GFP-infected cells (Fig. 4B). This shows that IL-10 production is pivotal to the functional impairment of CD4 T cell control by pUL11.

10.1128/mbio.02946-22.7 FIG S7 Expression of CMV-IL-10 in RPE cells. RPE cells were left uninfected (RPE) or infected with the HCMV Merlin GFP (HCMV WT) or HCMV Merlin dUL11 GFP (hcmv dul11) viruses for 6 days at an MOI of 1.0. The indicated volumes of lysates were used to detect the expression of CMV-IL-10 via immunoblotting, using a biotinylated anti-CMV-IL-10 antibody and Avidin-HRP for detection. Download FIG S7, TIF file, 0.1 MB. Copyright © 2022 Osanyinlusi et al. 2022 Osanyinlusi et al. https://creativecommons.org/licenses/by/4.0/ This content is distributed under the terms of the Creative Commons Attribution 4.0 International license.

### pUL11 affects both cytolytic and cytokine-dependent CD4 T cell control of HCMV.

CD4 T cell control of HCMV spread can occur via direct cytotoxicity targeted at the infected cells or by the secretion of effectors, including IFN-γ, by effector memory T cells (8). IL-10 has a range of direct, generally inhibitory, effects on CD4 T cells. In order to understand the effects of pUL11-induced IL-10 in more detail, we considered both direct cytolytic functions and the production of IFN-γ.

**FIG 5:**
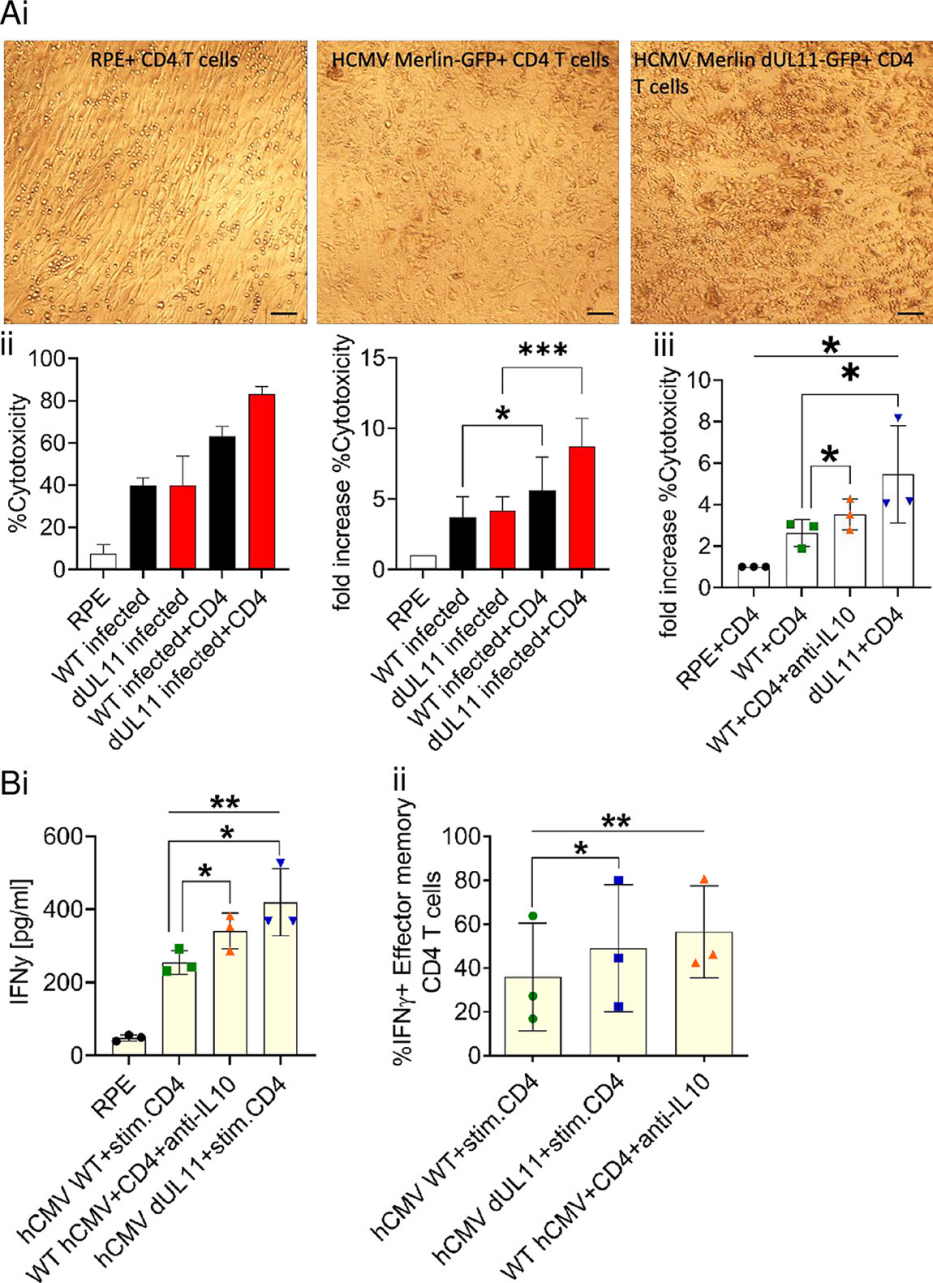
pUL11 affects both cytolytic and cytokine dependent CD4 T cell control of HCMV. RPE cells that were uninfected (RPE) or infected with HCMV Merlin GFP (WT) or HCMV Merlin dUL11 GFP (dUL11) were cultured with CD4 T cells that had been pre-stimulated with HCMV-infected cell lysate and anti-CD28 for 24 h in the presence or absence of an IL-10 blocking antibody (anti-IL-10) for 7 days. (A) (i) Light microscopic images from one representative experiment out of five, using T cells from five different CMV^+^ donors. Scale bar= 100 μm. (ii and iii) Cytotoxicity was determined via lactate dehydrogenase (LDH) release. The values from one representative donor and the fold increase over that from uninfected RPE cells with CD4 T cells from three experiments are shown, using T cells from three different CMV^+^ donors. (B) (i) Secreted IFN-γ was measured via ELISA, using T cells from three different CMV^+^ donors. (ii) CD45R0^+^ CCR7^–^ IFN-γ^+^ CD4 T cells were measured via flow cytometry. The percentages of IFN-γ expression for CD4 T cells from three different CMV^+^ donors are shown. Error bars represent the standard deviations of biological replicates. The statistical significance of the differences between groups was determined via one-way ANOVA.

We determined the effects of pUL11 and IL-10 on cytotoxicity visually and by using a LDH release assay (Fig. 5A). While the coculture of uninfected RPE cells with enriched CD4 T cells from HCMV seropositive donors induces only negligible degradation of the RPE cell monolayer, the HCMV Merlin GFP infection of the RPE cells induces cytolysis. This is substantially increased in cultures infected with HCMV Merlin dUL11 GFP in the absence of pUL11, again indicating that pUL11 reduces T cell function. The measured LDH is derived exclusively from lysed RPE cells; the pUL11 and lysate treatment of the T cells does not cause any LDH release (Fig. S5C). Infection of the RPE cells induces some cell lysis directly (in the absence of T cells), as shown in Fig. 5A. However, this background release of LDH does not differ between infections with HCMV Merlin GFP and infections with HCMV Merlin dUL11 GFP. Coculturing enriched CD4 T cells from HCMV seronegative donors with HCMV Merlin GFP-infected RPE cells did not induce cytolysis (Fig. S5A), indicating that the observed cytolysis is specific. Enriched CD4 T cells from HCMV seropositive donors were also unable to induce any substantial lysis of the uninfected RPE cells, even in the presence of IFN-γ which upregulates MHC class II expression in RPE cells, indicating that this effect is not due to nonspecific alloresponses (Fig. S5A). Blocking IL-10 increased the cytotoxicity in HCMV Merlin GFP-infected cocultures but not to the extent seen in the presence of HCMV Merlin dUL11 GFP infections, indicating that pUL11 also inhibits cytotoxicity by other, additional mechanisms.

Then, we considered the effects of pUL11 and IL-10 on effector memory IFN-γ secretion (Fig. 5B). The presence of HCMV Merlin GFP-infected RPE cells induces IFN-γ positive effector memory CD4 T cells, but the number of IFN-γ positive cells was markedly increased in infections with HCMV Merlin GFP dUL11 in the absence of pUL11. Similarly, the amount of secreted IFN-γ in the coculture supernatant, as measured by ELISA, was increased when pUL11 was absent from the virus, in comparison to that seen with HCMV Merlin GFP infections. Blocking IL-10 in HCMV Merlin GFP-infected cocultures led to an increase in both the number of IFN-γ secreting effector memory T cells and in the amount of secreted IFN-γ, and these were increased to similar levels to those observed in the HCMV Merlin GFP dUL11 cocultures.

These experiments show that pUL11 inhibits CD4 T cell control of HCMV spread by reducing both cytotoxicity and IFN-γ production, largely (but not entirely) via the effects of the induced IL-10 secretion.

### Signaling requirements of pUL11-dependent IL-10 induction.

**FIG 6:**
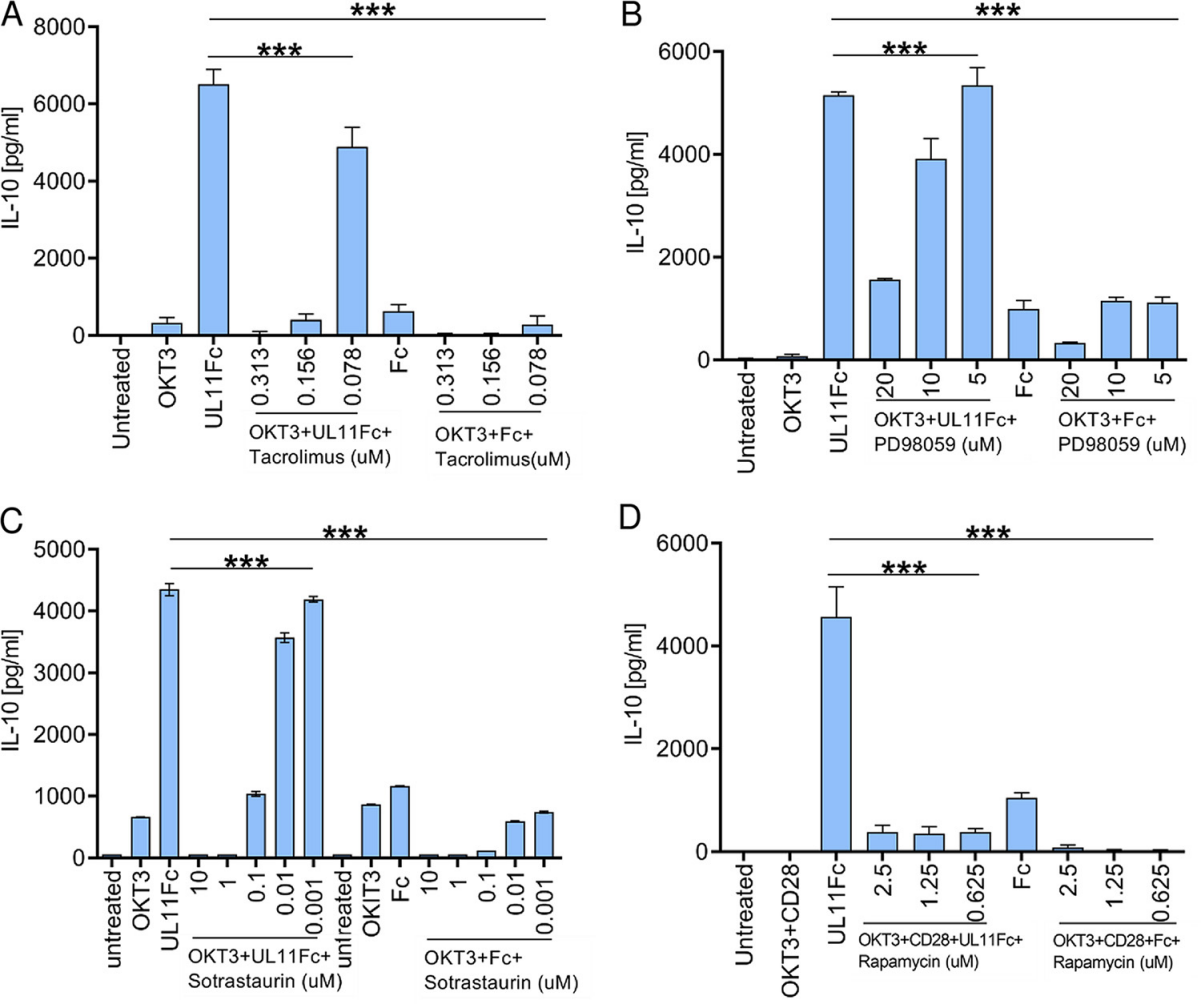
Signaling requirements of pUL11-dependent IL-10 induction. PBMCs (A–C) or enriched CD4 T cells (D) were untreated, stimulated via anti-CD3 (OKT3), or additionally stimulated with anti-CD28 (OKT3^+^CD28) in the presence or absence of Fc or UL11Fc and in the presence of a calcineurin inhibitor (tacrolimus) (A), a MAPK inhibitor (PD98059) (B), a PKC inhibitor (sotrastaurin) (C), and an mTOR inhibitor (rapamycin) (D) for 96 h. Secreted IL-10 was measured via ELISA. Error bars indicate the standard deviations of three biological replicates. A one-way ANOVA (A–D) was used to determine the differences between groups.

TCR signaling is the starting point for IL-10 induction. Downstream of the TCR, the pathway divides into four main branches; calcium-calcineurin (inhibited by tacrolimus), MEK 1 and 2 (inhibited by PD98059 and feeding into ERK1/2), PKC (inhibited by sotrastaurin), and mTOR (inhibited by rapamycin). The inhibition of each of these branches individually has been shown to inhibit T cell IL-10 production (39–42). We treated PBMC or CD4 T cells stimulated via the TCR with the addition of anti-CD28 to provide the necessary co-stimulation in the T cell cultures via pUL11 in the presence of inhibitors of each branch and showed that all four could block or reduce pUL11-dependent IL-10 induction without reducing cell viability (Fig. 6; Fig. S8). Therefore, all four branches are required for optimal IL-10 production, as has been shown for IL-10 secretion in T cells induced by standard methods, indicating that all of the strands of the TCR signaling network converge to result in the observed pUL11-induced functional changes to memory T cells.

10.1128/mbio.02946-22.8 FIG S8 Treatment of PBMCs with pathway inhibitors. PBMCs (A–C, E) or enriched CD4 T cells (D) were unstimulated (medium), stimulated via anti-CD3 (OKT3), or additionally stimulated with anti-CD28 (OKT3^+^CD28) in the presence of the Fc control (Fc) or UL11Fc and various concentrations of tacrolimus (A), PD98059 (B), sotrastaurin (C), rapamycin (D), or IL-2 block (E) as shown for 96 h at 370°C. Cellular viability was determined via MTT assay. Download FIG S8, TIF file, 0.9 MB. Copyright © 2022 Osanyinlusi et al. 2022 Osanyinlusi et al. https://creativecommons.org/licenses/by/4.0/ This content is distributed under the terms of the Creative Commons Attribution 4.0 International license.

**FIG 7:**
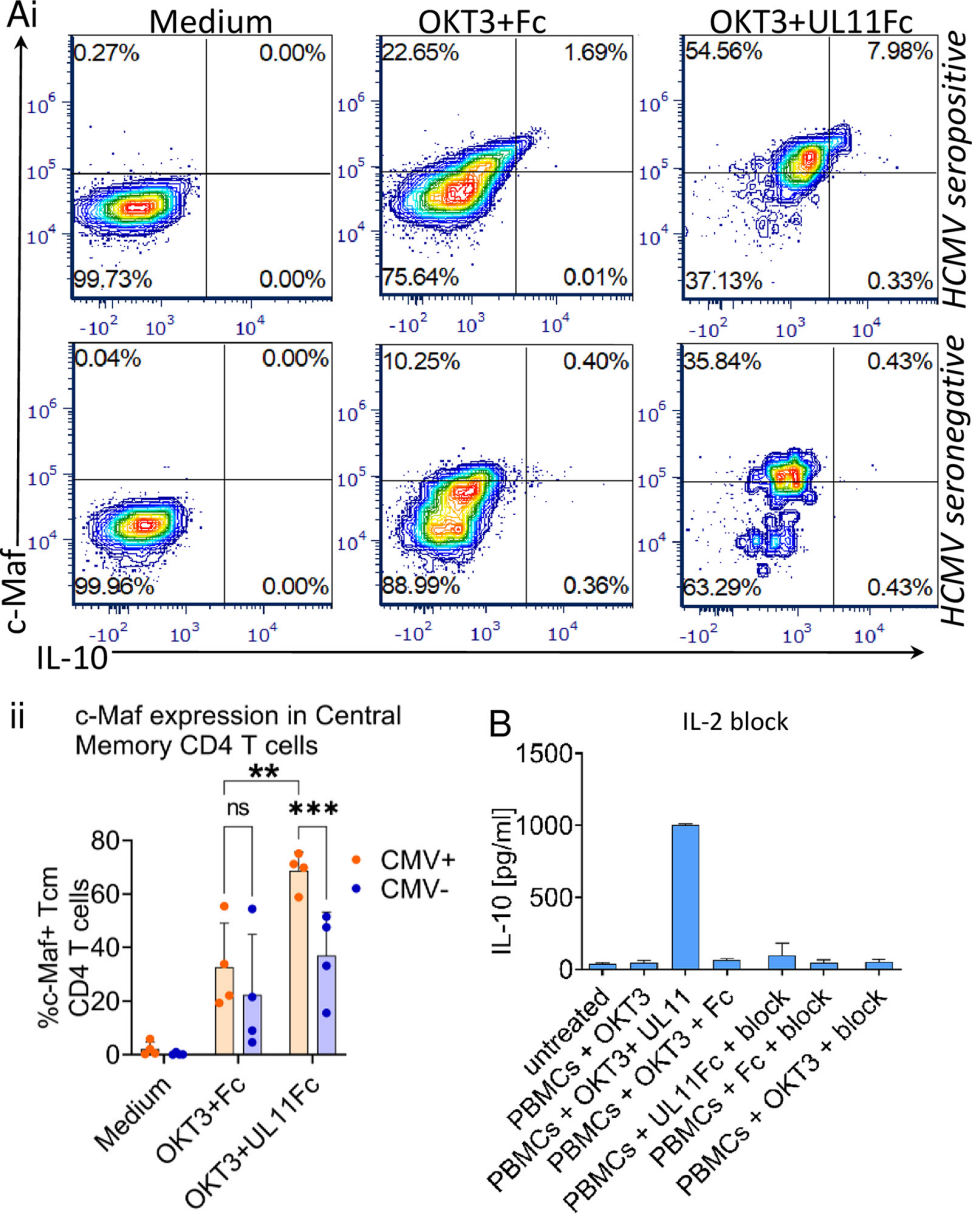
pUL11 upregulates c-Maf expression in memory CD4 T cells, and IL-2 signaling is required for IL-10 induction. PBMCs were unstimulated (medium) or stimulated via anti-CD3 (OKT3) with the Fc control (Fc) or pUL11Fc for 48 h. (A) c-Maf and IL-10 expression in CD3^+^ CD4^+^ CD45RO^+^ CCR7^+^ T cells was measured via flow cytometry. One representative experiment (i) as well as plots showing the mean percentage of live c-Maf^+^ central memory CD4 T cells from four CMV^+^ and four CMV^–^ donors (ii) are shown. Error bars indicate the standard deviations of biological replicates. (B) IL-2 inhibitors (block) (anti-IL-2, anti-IL-2 receptor [CD25], and anti-CD122), were added to the culture for 96 h. Secreted IL-10 was measured via ELISA. Error bars indicate the standard deviations of three biological replicates, using PBMC from three different donors, and *t* tests were used to determine the statistical significance of the differences between groups.

The transcription factor c-Maf is induced upon TCR stimulation and is then the subject of complex regulatory mechanisms. c-Maf directly induces IL-10 expression, both alone and in combination with other transcription regulators (43). We showed that the induction of c-Maf upon TCR stimulation is enhanced in pUL11 treated CD4 memory T cells and that the cells expressing the highest levels of c-Maf are the IL-10 producers (Fig. 7A). The pUL11-driven increase in c-Maf expression was particularly pronounced in cells from HCMV seropositive donors, which presumably underlies the higher capacity for pUL11 to induce IL-10 in these cells (Fig. 7A). We tested the effects of the four TCR signaling inhibitors on c-Maf induction and showed that while sotrastaurin and tacrolimus do inhibit c-Maf induction, PD98059 and rapamycin do not (Fig. S9). Although c-Maf has been described as being essential for IL-10 production, it seems that is not sufficient and that rapamycin and PD98059 inhibit other essential steps in the induction pathway in this setting.

10.1128/mbio.02946-22.9 FIG S9 Effects of pathway inhibitors on c-Maf expression in central memory CD4 T cells. Enriched CD4 T cells were unstimulated (medium) or stimulated via anti-CD3 (OKT3) and CD28 with the Fc control (Fc) or pUL11Fc and the indicated inhibitors for 48 h. c-Maf and IL-10 expression in CD45RO^+^ CCR7^+^ CD4 T cells was measured via flow cytometry. One representative experiment (i) and plots showing the mean percentage of live c-Maf^+^ central memory CD4 T cells from two CMV^+^ donors are shown. Error bars indicate the standard deviations of biological replicates. Download FIG S9, TIF file, 1.0 MB. Copyright © 2022 Osanyinlusi et al. 2022 Osanyinlusi et al. https://creativecommons.org/licenses/by/4.0/ This content is distributed under the terms of the Creative Commons Attribution 4.0 International license.

IL-2 signaling drives the expression of c-Maf (44). The use of IL-2 blocking antibodies (45) prevented the pUL11-dependent induction of IL-10 without affecting cell viability (Fig. 7B; Fig. S8E). Therefore, the induction of IL-10 by pUL11 seems to follow previously described patterns. We have previously shown that pUL11 treatment mediates the strength of signaling through the TCR via its interaction with CD45 but that it does not completely inhibit TCR signal transduction. Here, we see again that pUL11 tightly controls TCR signal strength and that events downstream of the TCR are required for its immunoregulatory functions.

## DISCUSSION

HCMV is capable of lifelong persistence in the host, despite robust immune responses. T cell responses are critical to the control of HCMV infections, yet they are unable to eradicate the virus due to multilayered immune evasion and manipulation by the virus. The virus has many ways of avoiding detection by T cells; antigen presentation is disabled both from the side of infected tissues and by the inhibition of key mechanistic features of antigen-presenting cells. The direct inhibition of T cell function via contact with infected cells has historically been observed, but the mechanisms involved are not yet fully clear (46, 47). Here, we describe a mechanism by which HCMV limits T cell control, not by evading recognition, but by an interaction with the CD45 phosphatase on the T cell surface. CD45 acts as a negative regulator of antiviral functions in this context, with some parallels to the receptors on NK cells that are also exploited by HCMV.

While CD8 T cells form a core of the immune defenses, HCMV has many strategies by which to reduce the MHC Class I-dependent antigen presentation on which CD8 T cell functions depend. It has recently been shown that an additional facet of immune defense against many viruses is MHC Class II-dependent CD4 T cells, which are important not only in terms of their helper functions but also as antiviral effectors in their own right. CD4 T cells can act by secreting antiviral cytokines, particularly IFN-γ, which induces antiviral effects in neighboring cells by suppressing viral replication, or by cytolytic functions (48). Cytotoxic CD4 T cells have been described in response to several viruses, including influenza, HIV, hepatitis D, and vaccinia (49–51). However, it is in herpesvirus infections that CD4 CTLs are perhaps best known, as they have been observed in response to all three families of human herpesviruses (52–54). CD4 effector T cells in HCMV infections act via both cytokine-dependent and cytolytic mechanisms (7, 55, 56). Cytotoxic CD4 T cells have been isolated directly from HCMV patients, and IFN-γ-secreting CD4 effector memory T cells are essential for the effective control of HCMV infections (7, 29).

CD4 T cells are important for both primary and recall responses against HCMV, but the recall responses are more extensive and potent (55). Central memory T cells are readily able to proliferate and to secrete IL-2 and a small range of effector cytokines (57), thus further inducing proliferation. The proliferating cells then differentiate into effector memory T cells, which are able to provide extended, direct antiviral functions and help to other sections of the immune system. In stem cell and renal transplant recipients, IFN-γ producing CD4 memory T cell numbers correlate with the control of HCMV (29, 58). Therefore, the CD4 T cell memory compartment would seem to be a good target for viral immunomodulation. We show that pUL11 specifically affects these cells, induces IL-10 secretion in central memory cells, and ultimately reduces IFN-γ secretion and cytotoxicity in the effector memory compartment.

CD4 T cells respond to antigens presented via MHC class II. The classical view of MHC class II-dependent antigen presentation is that extracellular proteins are internalized and cleaved in the endosomal/lysosomal compartment, where they encounter MHC class II molecules. Then, bound to MHC class II, they enter the plasma membrane, from where they can be presented to CD4 T cells. Internal proteins can, however, also be presented by MHC class II, and they account for up to 30% of presented peptides (59). HCMV and other herpesviruses, which assemble viral particles in the TGN/endosomes and thus have many structural proteins available for processing, may be particularly susceptible to this type of MHC class II presentation, and, therefore, to CD4 T cell responses (9).

HCMV uses many routes to evade MHC Class II-dependent antigen presentation. During an HCMV infection, endogenous MHC II expression is downregulated (60), IFN-γ dependent inducible expression of MHC II is inhibited (61), the assembly of the peptide-MHC II complex is blocked (62), the localization of MHC II is disrupted (63), and the complex is targeted for degradation (64). Despite all of these mechanisms, however, MHC II expression is not completely disrupted, as we also show in a proportion of infected RPE cells, and antigen presentation to CD4 T cells by infected macrophages and glial cells has been observed (9, 65).

MHC class II is constitutively expressed on “professional” antigen-presenting cells, such as DCs and macrophages. It can also be induced by exposure to IFN-γ in a range of other cell types, including epithelial cells, endothelial cells, and fibroblasts. Epithelial cells typically have reduced costimulatory molecule expression in comparison to their professional counterparts, meaning that although antigen presentation is possible, a reduced or altered range of CD4 T cell responses is induced (66). While memory responses can be triggered, for example, following recall stimulation, the functional priming of naive T cells may not be possible (67). MHC class II-dependent epithelial cell antigen presentation to CD4 effector T cells *in vivo* has been described for several viruses (68, 69).

HCMV infects a wide range of cell types *in vivo*. Epithelial cells are the main cell type to be infected in the lungs, GI tract, and kidneys, and the epithelial cells of the rhinopharynx are the first cells to be infected following oral transmission (10). HCMV retinitis is a frequent outcome of HCMV infection in people with reduced T cell function and is typified by progressive lesions that can result in blindness. Infection of RPE cells is crucial to the development of the disease (11). Because epithelial cells are important to the course of an HCMV infection *in vivo*, it is important to understand how infection is controlled in these cells. Therefore, we chose RPE cells as a relevant cell type with which to study CD4 T cell control of HCMV spread.

CD4 T cells have been shown to control HCMV infections *in vitro* in dendritic cells with constitutive MHC class II expression (8). We show here that CD4 T cell control of HCMV in infected epithelial cells is also possible. We describe a new coculture system, using primary T cells and epithelial cells infected with a GFP-expressing derivate of HCMV, in which the roles of T cell subsets can be investigated. Memory CD4 T cells from HCMV seropositive donors can be primed using APCs loaded with lysate from HCMV infected cells and can then respond to infected RPE cells. Infection with HCMV upregulates MHC class II expression on the RPE cells, and this allows for sufficiently robust antigen presentation to initiate CD4 T cell control of the infection. As was seen with the HCMV-infected DCs, CD4 T cells control infection in RPE cells via both cytokines (IFN-γ) and cytolytic mechanisms.

Although viral spread in epithelial cells is limited by CD4 T cells, it is not completely eliminated. We show that this control is greatly impaired by the interactions of the pUL11 glycoprotein with T cells. Investigations into the mechanisms by which cytomegalovirus and other herpesviruses inhibit CD4 T cell responses have mostly focused on MHC class II inhibition in infected cells, rather than on the effects on the T cells themselves. The impairment of uninfected NK cell functions via direct contact with HCMV proteins on infected cells has been described in detail, and this includes mechanisms by which NK cell responses are directly downgraded in addition to the evasion of detection (70). We now describe a means by which pUL11 can also act on CD4 T cells directly, altering their functionality and resulting in impaired anti-HCMV responses.

pUL11 is expressed on the surfaces of HCMV-infected cells and interacts with the tyrosine phosphatase CD45 on the surfaces of neighboring uninfected lymphocytes, including CD4 T cells (24). CD45 is essential for signaling through the TCR, with concentration dependent effects that allow it to either enhance or inhibit the strength of ongoing signals via its ability to dephosphorylate two regulatory tyrosine residues in the src kinase Lck (71). Changes in T cell signaling strength can underlie a diverse range of functions, and the ligation of CD45 by monoclonal antibodies and lectins results in effects including IL-10 secretion and the induction of a suppressive function in T cells (26, 27, 72). A CD45 mAb has shown promise in reducing transplant rejection due to these anti-inflammatory effects (26). Although the lectins that interact with CD45 typically have additional binding partners, the similarity of the lectin, mAb, and pUL11-induced effects indicates that IL-10 induction in T cells can be driven by CD45 ligation in combination with TCR stimulation. The observation that CD45 acts as an inhibitory receptor to regulate antiviral T cell functions is, however, novel. We have previously shown that pUL11 also has the ability to induce T cell IL-10 secretion. IL-10 acts on antigen-presenting cells, impairing their ability to activate T cells, and also acts directly on T cells, displaying direct, suppressive effects on CD4 T cells and thereby reducing proliferation, cytotoxicity, and IFN-γ secretion (16, 17). Increased IL-10 production during HCMV infections in transplant patients is linked with HCMV disease (14). HCMV also expresses several different isoforms of a homologue of IL-10 (which is not affected by pUL11). These cmvIL-10 proteins exert immunosuppressive functions via their interactions with the IL-10 receptor and are expressed during both lytic and latent infections, indicating the importance of this pathway for the virus (73). IL-10-producing CD4 T cells that are specific for both latently and lytically expressed proteins have been described in blood and mucosal tissue samples from HCMV seropositive individuals, with the secreted IL-10 having the ability to inhibit polyclonal T cell proliferation (8, 22, 74). In MCMV infections, peripheral IL-10^+^ CD4 T cells impair the control of both chronic and acute infections in mucosal tissues (22, 75). IL-10 induction during HCMV infections may also have wider effects on immune responses. IL-10 has complex effects on the regulation of different components of the immune system, meaning, for example, that the immune control of other infections, tumor development, or graft versus a host disease in transplant recipients could potentially be influenced.

Here, we identify the source of pUL11-induced IL-10 to be the CD4 central memory T cells and demonstrate that the presence of pUL11, either as a purified protein or expressed on the surface of HCMV infected cells, is sufficient to reduce the ability of CD4 T cells to control viral spread. The presence of IL-10 then impairs the ability of effector T cells to secrete IFN-γ and also reduces their cytotoxic function (i.e., it affects both aspects of CD4 T cell antiviral functions). pUL11 also exhibits an IL-10 independent effect to reduce cytotoxicity. Targeting the central memory CD4 T cell subset is presumably advantageous to the virus, as it ensures the earliest possible production of IL-10, meaning that the effector memory T cells are, from the onset of maturation, surrounded by IL-10 and subjected to its suppressive properties.

How CD45 is linked to IL-10 production is not yet understood. The signaling intermediates downstream of the TCR, which have been shown in other settings to be important for IL-10 induction, are similarly important here, as are the induction of c-Maf and the requirement for IL-2. Therefore, the pathway to IL-10 induction contains familiar components.

In conclusion, we have shown that HCMV-infected epithelial cells can be targets for CD4 T cells and that the viral spread in epithelial cells can be controlled by both interferon gamma and cytolytic effects of CD4 T cells, despite the presence of genes in HCMV that are able to reduce MHC class II expression. CD4 T cell responses can be directly impaired by HCMV via the interaction between pUL11 on the surfaces of infected cells and the T cell CD45, which represents a new means of immunomodulation by the virus, as the manipulation of T cell function has previously been largely discussed in terms of immune evasion via the reduction of MHC expression on infected cells. This ability of pUL11 to act on T cell function may have relevance to antiviral therapies, vaccine development, and the development of treatments to reduce pathologically excessive T cell functions.

## MATERIALS AND METHODS

### Ethics statement.

Human blood cells from healthy volunteer blood donors were provided by the Institute of Transfusion Medicine, Hannover Medical School. The HCMV serostatus (positive or negative) and the HLA type of each donors was available. All materials and data were analyzed anonymously. The ethics committee of Hannover Medical School approved the use of human blood cells.

### Cell preparation and culture.

The HLA typing of blood cells and of the RPE cell line was performed in the Institute of Transfusion Medicine, Hannover Medical School, using high resolution, targeted next-generation sequencing (NGS) of the HLA region as previously described (76).

Human peripheral blood mononuclear cells (PBMCs) were isolated via Biocoll (Merck Millipore) density gradient centrifugation. Aliquots were stored at −80°C until needed. T cells (CD4 or CD8) were isolated from PBMC via negative selection using magnetic beads (BD IMag Human CD4 or Human CD8 T Lymphocyte Enrichment Set-DM, Becton, Dickinson). Both PBMCs and T cells were cultivated in RPMI medium supplemented with 10%FCS, 1% penicillin/streptomycin, 2 mM l-glutamine, and 1 mM sodium pyruvate (ThermoFisher Scientific). The cells were incubated for at least 2 h or overnight before use.

Retinal pigmented epithelial (hTERT-RPE-1) cells (Clontech) were grown in Dulbecco’s Modified Eagle Medium-Nutrient Mixture F-12 (DMEM-F12, ThermoFisher) supplemented with 10% FCS, 1% penicillin/streptomycin, and 2 mM l-glutamine. In order to evaluate the MHC Class II expression on the RPE cells, 5 μg/mL recombinant human IFN-γ (Immunotools) were added to the culture medium for the indicated times.

### Protein production.

Fc protein production has been described previously (24, 32). Briefly, the predicted extracellular domain of pUL11 from the TB40 strain of HCMV, fused to the human IgG1 Fc domain (UL11Fc) and to this Fc domain alone as a control (Fc), was expressed from retrovirally transduced 293T cells. The predicted extracellular domains of pUL11 from the Merlin and Toledo strains of the virus, fused to the human IgG1 Fc domain, were expressed from adenovirally transduced RPE cells. All of the Fc proteins were purified by the Fc domain via protein A affinity chromatography.

### Stimulation of T cells.

1 × 10^5^ PBMCs or T cells were incubated in 96-well Maxi-Sorb plates (ThermoFisher) with 1 μg anti-CD3 (OKT3, purified from hybridoma supernatant) and, where indicated, 100 nM UL11Fc or Fc control preadsorbed onto the surface. For the T cells, 2 μg/mL of anti-CD28 (Clone L293, BD Pharmingen) were added to the medium. To generate the CMV-specific responses, 100 μg of HCMV-infected cell lysate (antibodies-online GmbH) was adsorbed onto the plate, and anti-CD28 (2 μg/mL) was added to the medium.

### Cytokine secretion.

Secreted IL-10 or IFN-γ in the culture medium was measured via ELISA (ELISA MAX Standard Set Human IL-10, or ELISA MAX Deluxe Set Human IFN-γ, BioLegend, Koblenz, Germany), according to the manufacturer’s instructions.

### Viruses.

The two viruses used in this study, HCMV Merlin GFP and HCMV Merlin dUL11 GFP, were kindly provided by Martin Messerle. HCMV Merlin GFP was based on the bacterial artificial chromosome (BAC)-cloned genome of HCMV Merlin-UL128^TB40^, and it contains a frameshift in RL13, and, after UL122, an IRES and the GFP encoding gene (77, 78). HCMV Merlin dUL11 GFP has a deletion of the UL11 open reading frame (ORF) and a substitution of the Gaussian luciferase ORF (described as the HCMV HM11DL mutant in [32]). Both viruses grow as cell-associated viruses in RPE cells grown in Dulbecco’s Modified Eagle Medium-Nutrient Mixture F-12 (DMEM-F12, ThermoFisher) supplemented with 10% FCS, 1% penicillin/streptomycin, and 2 mM l-glutamine. The viruses were maintained in culture via the addition of uninfected RPE cells to infected cells at a 1:1 ratio. New infections were initiated in RPE via the addition of stocks of both viruses at the desired multiplicity of infection (MOI). GFP expression was quantified via flow cytometry for the approximate determination of the infection rates. For the coculture experiments 7.5 × 10^4^ or 4.5 × 10^4^ PBMCs or T cells were incubated in triplicates in 96-well Maxi-Sorb plates with the described pretreatment additions for 24 h. RPE cells, infected with HCMV at 15 to 20%, as determined by the flow cytometry measurements of GFP where indicated, were also incubated in 96-well Maxi-Sorb plates for 24 h in DMEM F-12 medium, after which the medium was changed. PBMCs or T cells were then removed from the plate via vigorous pipetting and added to the RPE cells at an effector-to-target cell ratio of 3:1. The cocultures were maintained for 7 days.

### Virus stock production.

HCMV Merlin GFP or HCMV Merlin dUL11 GFP viruses were grown via the addition of infected RPE cells to uninfected RPE cells, and they were left for 7 to 10 days until an almost 100% cytopathic effect was reached. Whole cells and media in flasks were collected and centrifuged at 3,500 × *g* for 45 min at 4°C to remove cellular debris. The supernatants were thereafter subjected to ultracentrifugation at 62,808 × *g* for 1 h at 4°C. Virus titration was done in human foreskin fibroblast (HFF) cells cultured in Dulbecco’s Modified Eagle Medium (DMEM) (ThermoFisher) supplemented with 10% FCS, 1% penicillin/streptomycin, and 2 mM l-glutamine in 48-well plates at 37°C for up to 10 days, after which the fluorescence microscopic quantification of the green cells or plaque assay was performed.

### Flow cytometry and FACS sorting.

Cells were characterized by both extracellular and intracellular labeling. Live cells were detected via the exclusion of dye (Zombie Aqua Fixable Viability Kit, BioLegend). The T cell surface expression of CD3 (APC anti-human CD3, clone UCHT1, BioLegend, or FITC anti-human CD3, Clone OKT3, BioLegend), CD4 (FITC anti-human CD4, clone A161A1, BioLegend), CD45RA (FITC anti-Human CD45RA Clone HI100, BD or Brilliant Violet 510 anti-human CD45RA, Clone H100, BioLegend), CD45RO (APC anti-Human CD45RO Clone UCHL1, BD or FITC anti-Human CD45RO, Clone UCHL1, Biolegend, or PE Mouse Anti-Human CD45RO Clone UCHL1, BD), CD62L (PerCP/Cyanine5.5 anti-human CD62L, Clone DREG-56, BioLegend), CCR7 (PE/Cyanine7 anti-human CD197 [CCR7], Clone G043H7, BioLegend, or Pacific Blue anti-human CD197 [CCR7] Clone G043H7, BioLegend), CD127 (Pacific Blue anti-human CD127 [IL-7Rα], Clone A019D5, BioLegend), CD27 (FITC anti-human CD27, Clone 0323- BioLegend), and CD4 (PE/Cyanine5.5 anti-human CD4 Antibody, Clone OKT4, Biolegend) was measured. The RPE cell surface expression of MHC class II was measured using anti-HLA-DR APC (Clone LT-DR, Immunotools). The T cell intracellular expression of IL-10 (IL-10 PE, Clone JES3-19F1, BioLegend or anti-human IL-10 APC, Clone JES3-19F1, BioLegend), IL-2 (APC anti-human IL-2, Clone MQ1-17H12, BioLegend), c-Maf (PE anti-c-Maf Clone T54-853, BD), and IFN-γ (APC anti-human IFN-γ Antibody Clone 4S.B3, Biolegend) was measured.

For extracellular staining, the cells were incubated with antibody for 30 min and washed twice with FACS buffer (1× Dulbecco PBS [Cytogen] containing 2 mM EDTA).

For intracellular staining, T cells were fixed and permeabilized with fixation and permeabilization wash buffers (BioLegend), according to the manufacturer’s instructions. Then, they were incubated with antibody for 20 min and washed twice with permeabilization wash buffer.

For intracellular cytokine staining (IL-10, IL-2 and IFN-γ), brefeldin A (10 μM, for IFN-γ), monensin (4 μM, for IL-10), or a combination of the two (for IL-10 and IL-2 or IL-10 and c-Maf co-staining) were added to the culture for the last 6 h. Measurements were acquired with either a Cytoflex Beckman Coulter or a FACS Canto flow cytometer.

To separate the CD4 T cells into naive and memory populations, the cells were labeled as described above to detect the surface expression of CD45RA and CD45RO (FITC anti-Human CD45RA Clone HI100-RUO, and APC anti-Human CD45RO Clone UCHL1-RUO [BD]), and they were sorted accordingly. To further separate the CD4 T cell central and effector memory populations, the cells were additionally labeled with MAbs for CD62L and CCR7 (PerCP/Cyanine5.5 anti-human CD62L, Clone DREG-56, BioLegend) and PE/Cyanine7 anti-human CD197 (CCR7) Clone G043H7 (BioLegend) before sorting. Sorting was performed using a BD FACSAria III Cell Sorter.

### Microscopy.

Light and fluorescent images were taken at 10× magnification with a Nikon Eclipse TS100. The images were processed using Image J.

### Cytotoxicity assay.

T cell cytotoxicity was determined using the lactate dehydrogenase (LDH) assay (Abcam), following the manufacturer’s instructions. Briefly, 1.5×10^4^ RPE cells and 7.5×10^4^ T cells were incubated together in 96-well plates for 7 days. The plates were then centrifuged at 600 g for 10 min to clear the supernatant, and 10 μL of the supernatant was used for the colorimetric assay. To calculate the percentage cytotoxicity, the background absorbance of the medium at 450 nm was first subtracted. The following equation was then used:

Cytotoxicity % = (Test sample − low control)/(High control − low control) × 100

Where low control = e.g. uninfected RPE  +  T cells and high control = lysed RPE cells

### Pathway inhibition.

PBMCs or CD4 T cells were pretreated as described and incubated for 96 h with the addition of the indicated concentrations of pathway inhibitors: for mTOR, rapamycin (Sigma-Aldrich); for calcineurin, tacrolimus (Hycultec GmbH); for PKC, sotrastaurin (Hycultec GmbH); and for MAPK, PD98059 (Hycultec GmbH). Cellular viability was determined with a 3-(4,5-dimethylthiazol-2-yl)-2,5-diphenyltetrazolium bromide (MTT) assay (Sigma-Aldrich), following the manufacturer’s instructions. To block IL-2 and IL-10 signaling, the following antibodies were added to the culture medium: for IL-2, anti-IL-2, clone BG5 (Antibodies Online), anti-IL-2 receptor, clone basiliximab (Antibodies Online) and anti-CD122 (BD Pharmingen); for IL-10, anti-human IL-10 (Biolegend).

### Detection of cmvIL-10 via immunoblotting.

RPE cells (1.2 × 10^5^) were infected with HCMV Merlin GFP or HCMV Merlin dUL11 GFP at a MOI of 1.0. After 6 days in culture, the cells were trypsinized and washed with ice-cold PBS before lysis in 200 μL 5× SDS-PAGE loading buffer (300 mM TRIS-HCl, 10%SDS, 0.1%bromophenol blue, 50% glycerol, 300 mM β-mecaptoethanol). The lysates were separated via SDS-PAGE before transfer onto a nitrocellulose membrane. The membranes were blocked with Roti block (Roth), and cmvIL-10 was detected using Viral HCMV IL-10 Biotinylated Antibody clone BAF117 (R&D Systems) and Avidin-HRP (Biolegend) prior to visualization using the Super Signal West Femto Maximum Sensitivity Kit (Thermo Scientific).

### Statistical analysis.

GraphPad Prism version 9 was used for the statistical analyses. Analyses of variance (ANOVA) and *t* tests were used to determine differences between groups, considering a *P* value of <0.05 to be indicative of a statistically significant result. *, *P* < 0.05; **, *P* < 0.005; ***, *P* < 0.0005.
