# Supplementary figures and images for: Human Cytomegalovirus pUL11, a CD45 Ligand, Disrupts CD4 T Cell Control of Viral Spread in Epithelial Cells
Source: mBio. 2022 Nov 29;13(6):e02946-22. doi: 10.1128/mbio.02946-22 (PMC9765415; doi:10.1128/mbio.02946-22)

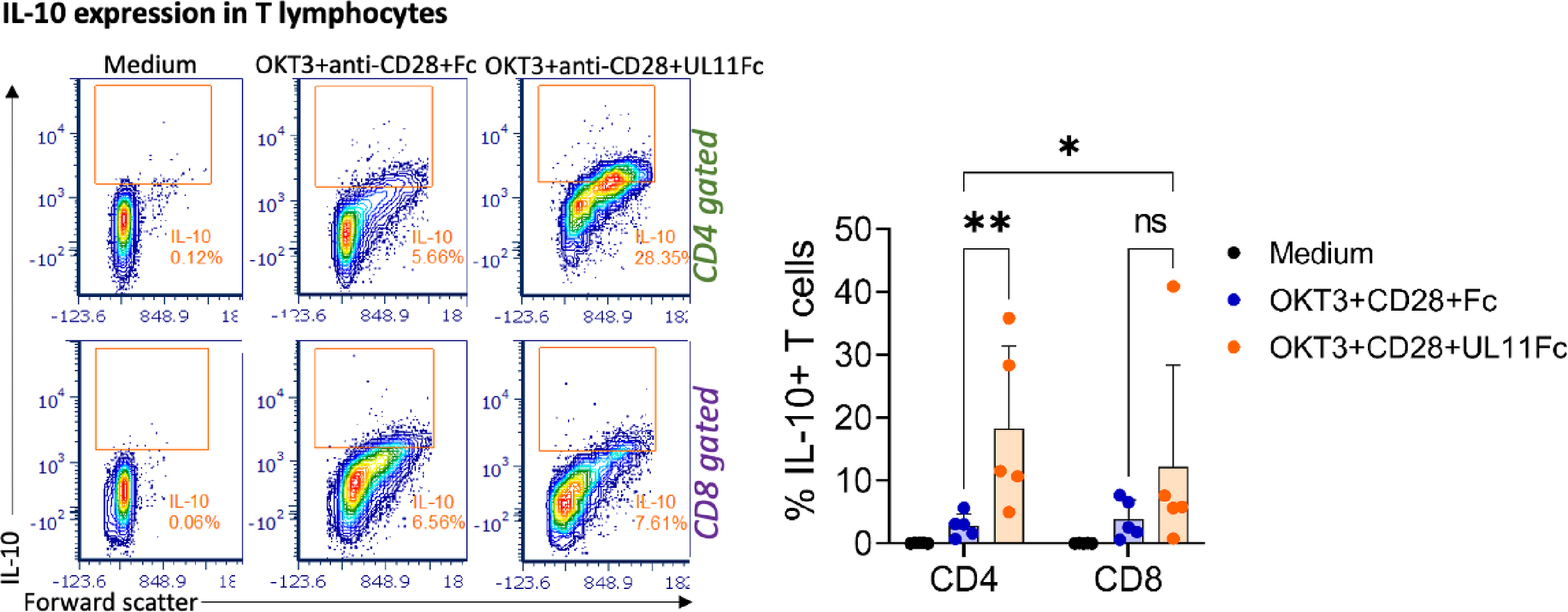

Supplement: FIG S1 [file mbio.02946-22-s0001.tif]

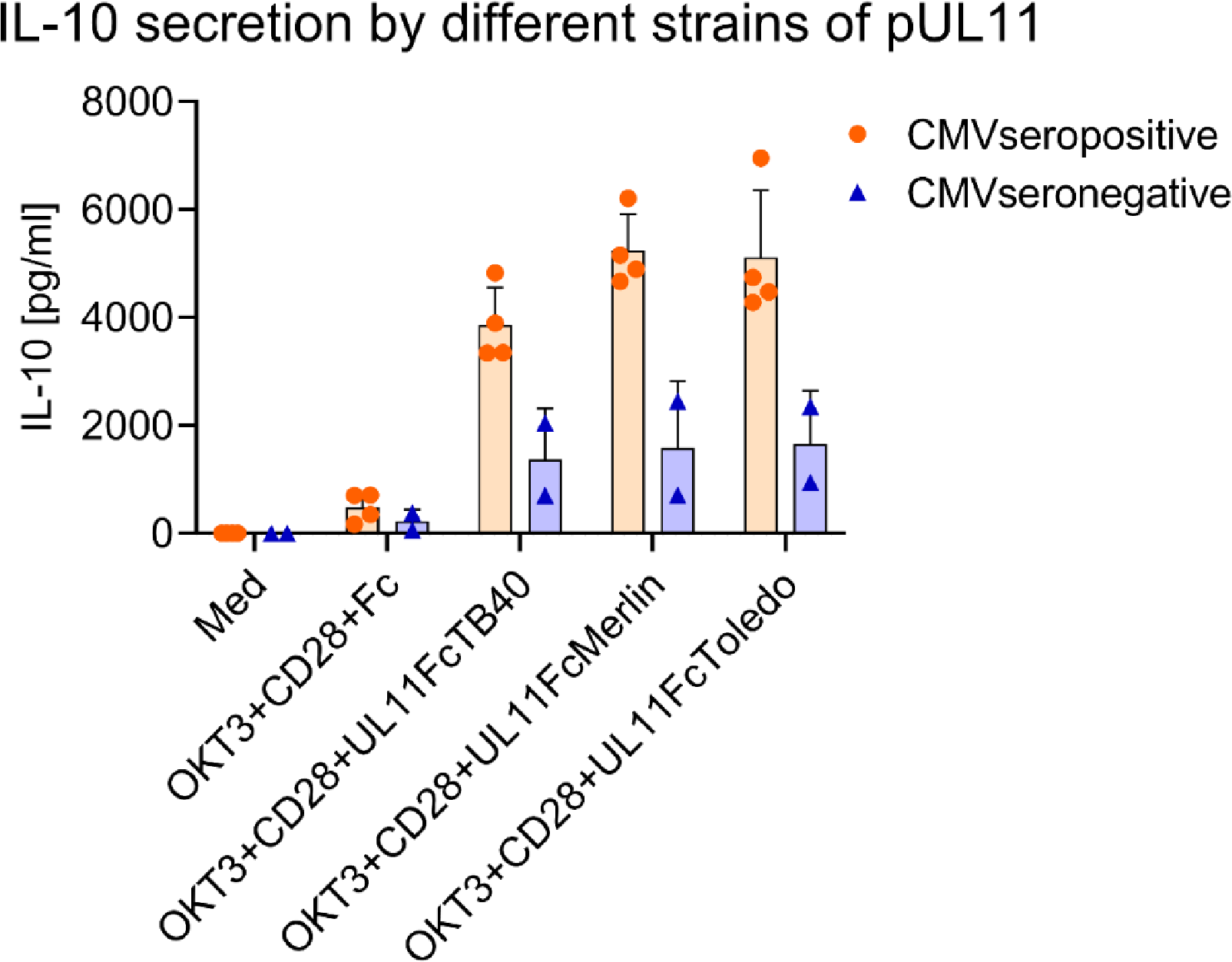

Supplement: FIG S2 [file mbio.02946-22-s0002.tif]

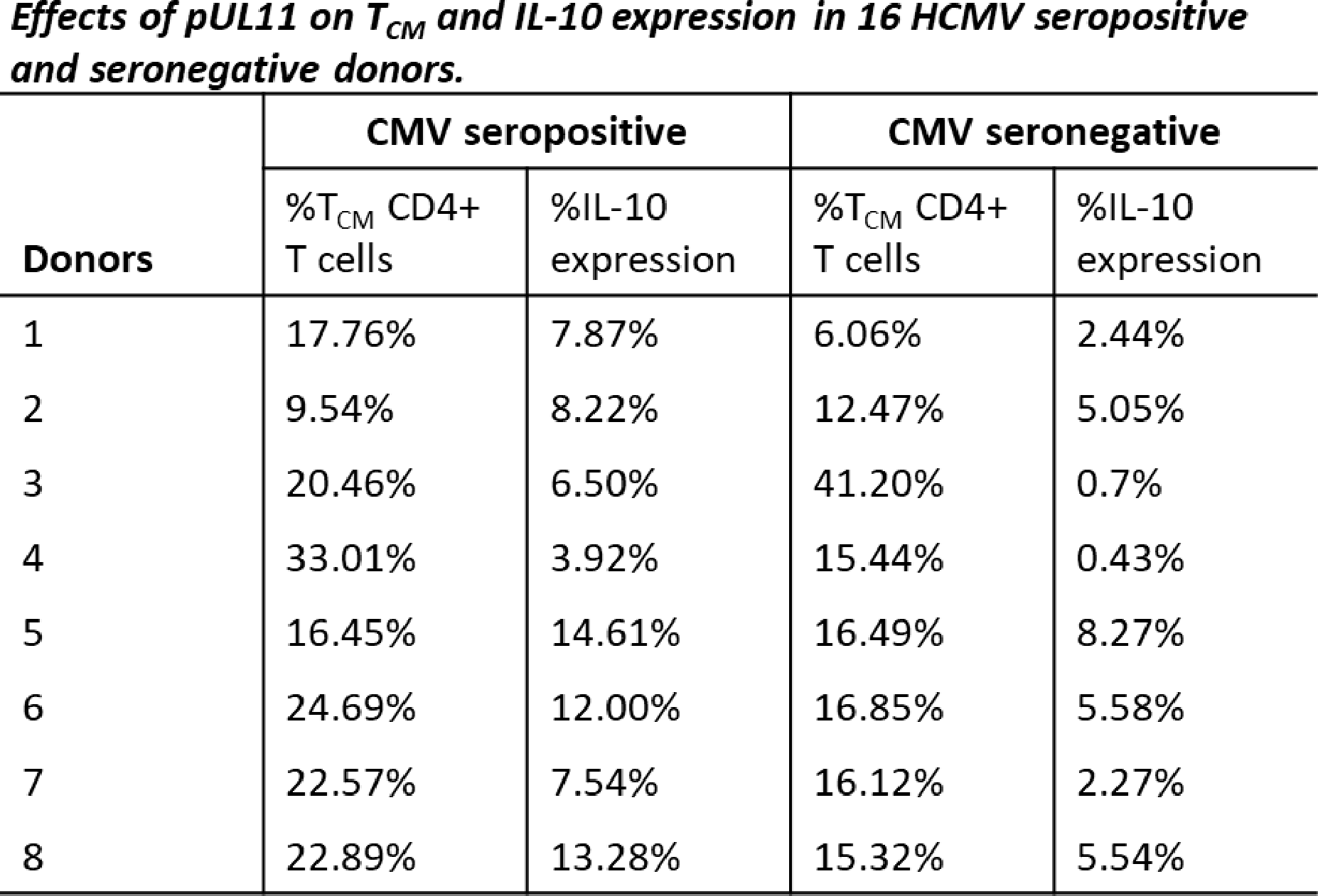

Supplement: TABLE S1 [file mbio.02946-22-s0010.tif]

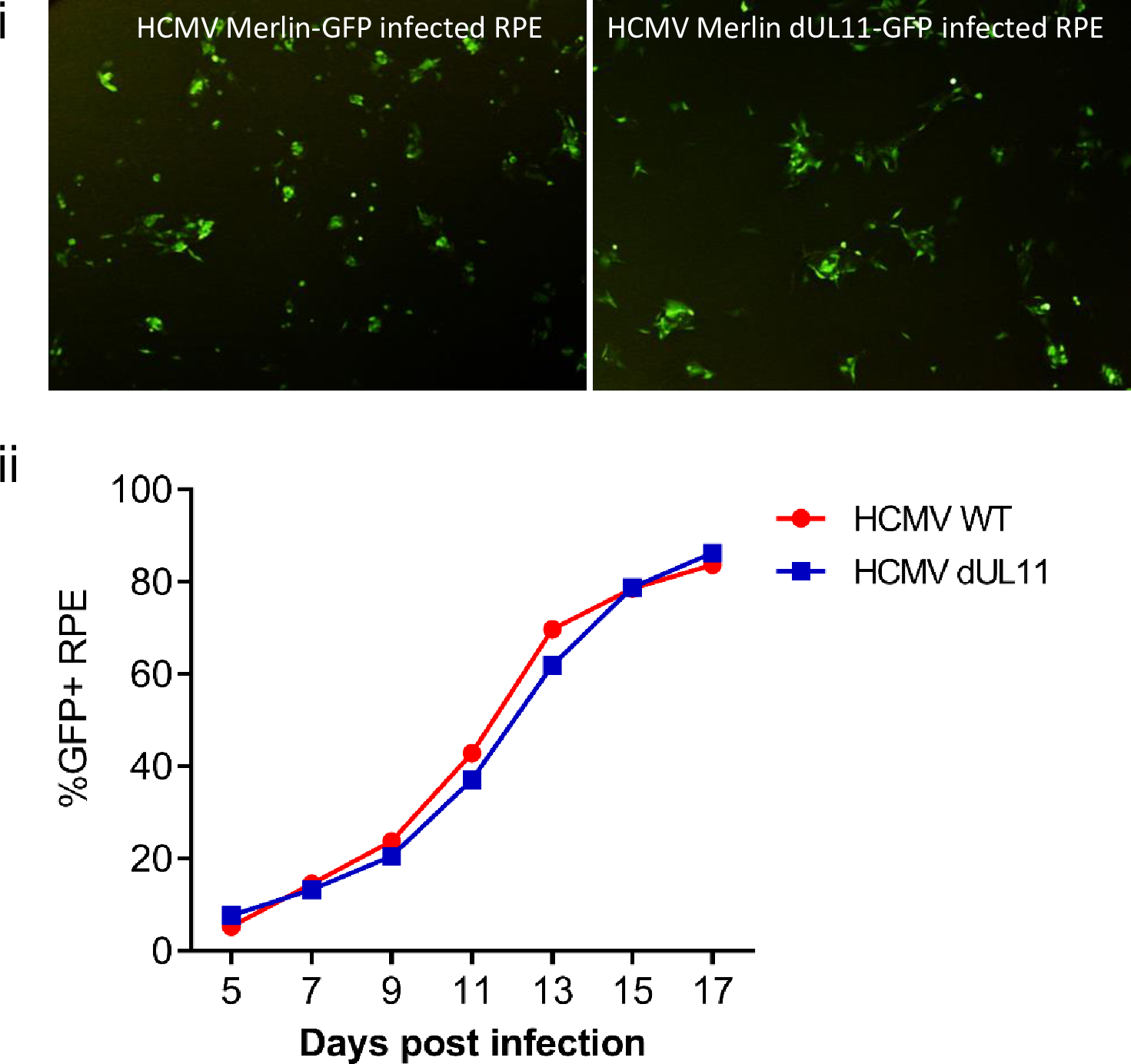

Supplement: FIG S3 [file mbio.02946-22-s0003.tif]

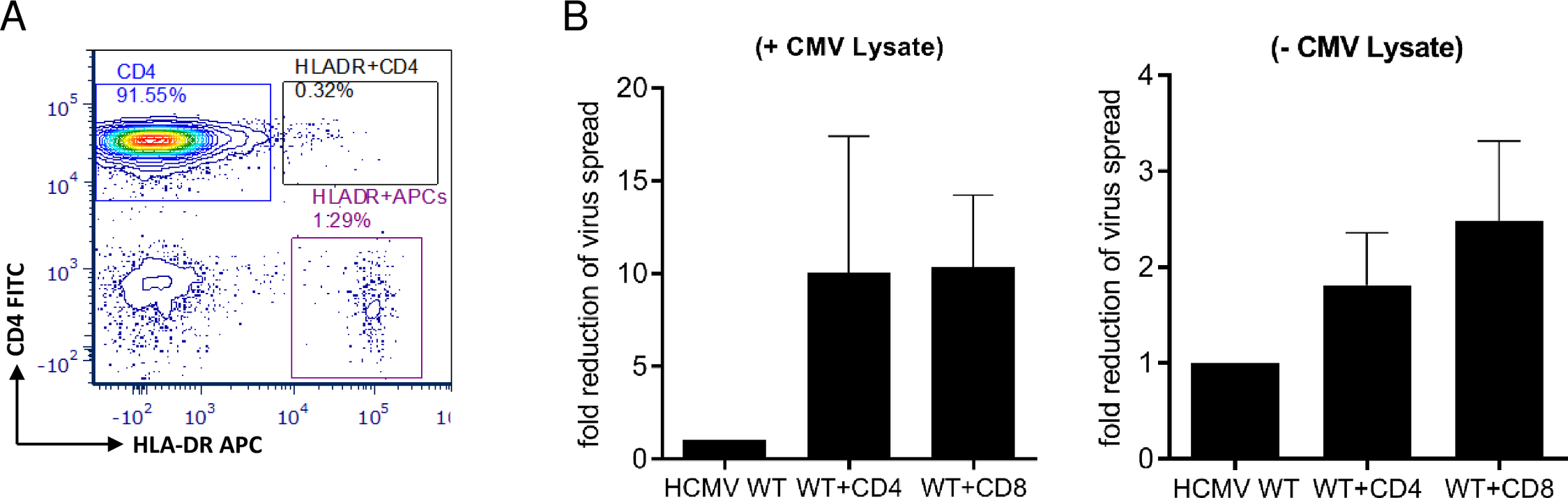

Supplement: FIG S4 [file mbio.02946-22-s0004.tif]

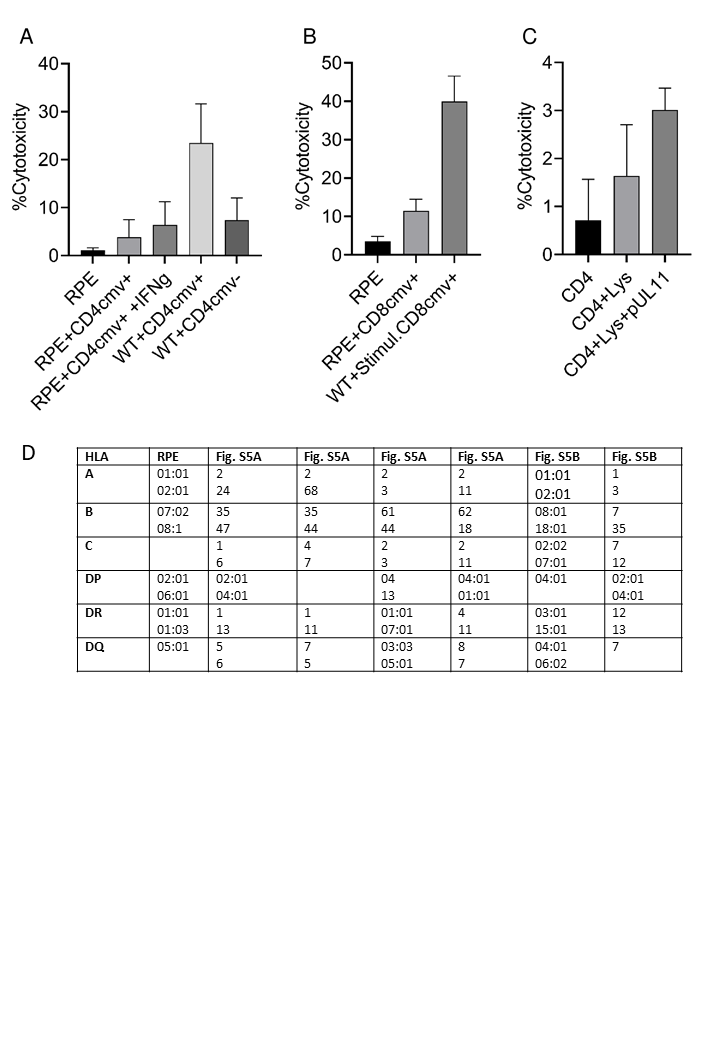

Supplement: FIG S5 [file mbio.02946-22-s0005.tif]

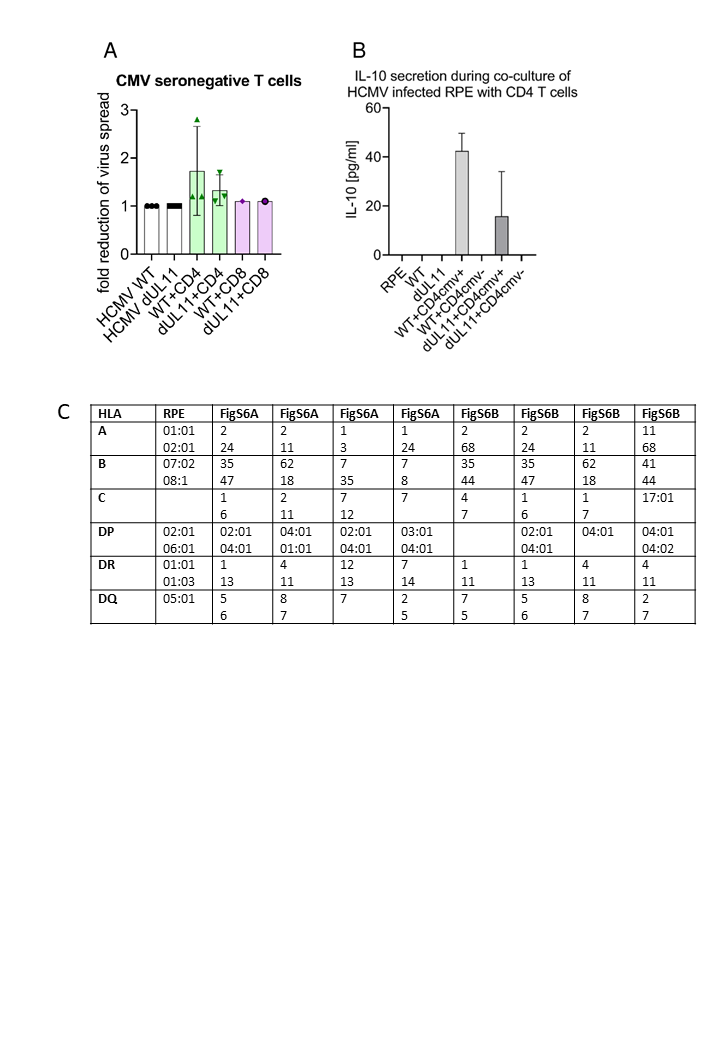

Supplement: FIG S6 [file mbio.02946-22-s0006.tif]

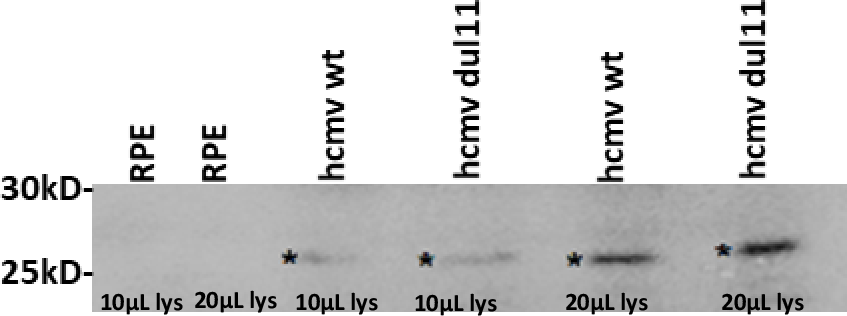

Supplement: FIG S7 [file mbio.02946-22-s0007.tif]

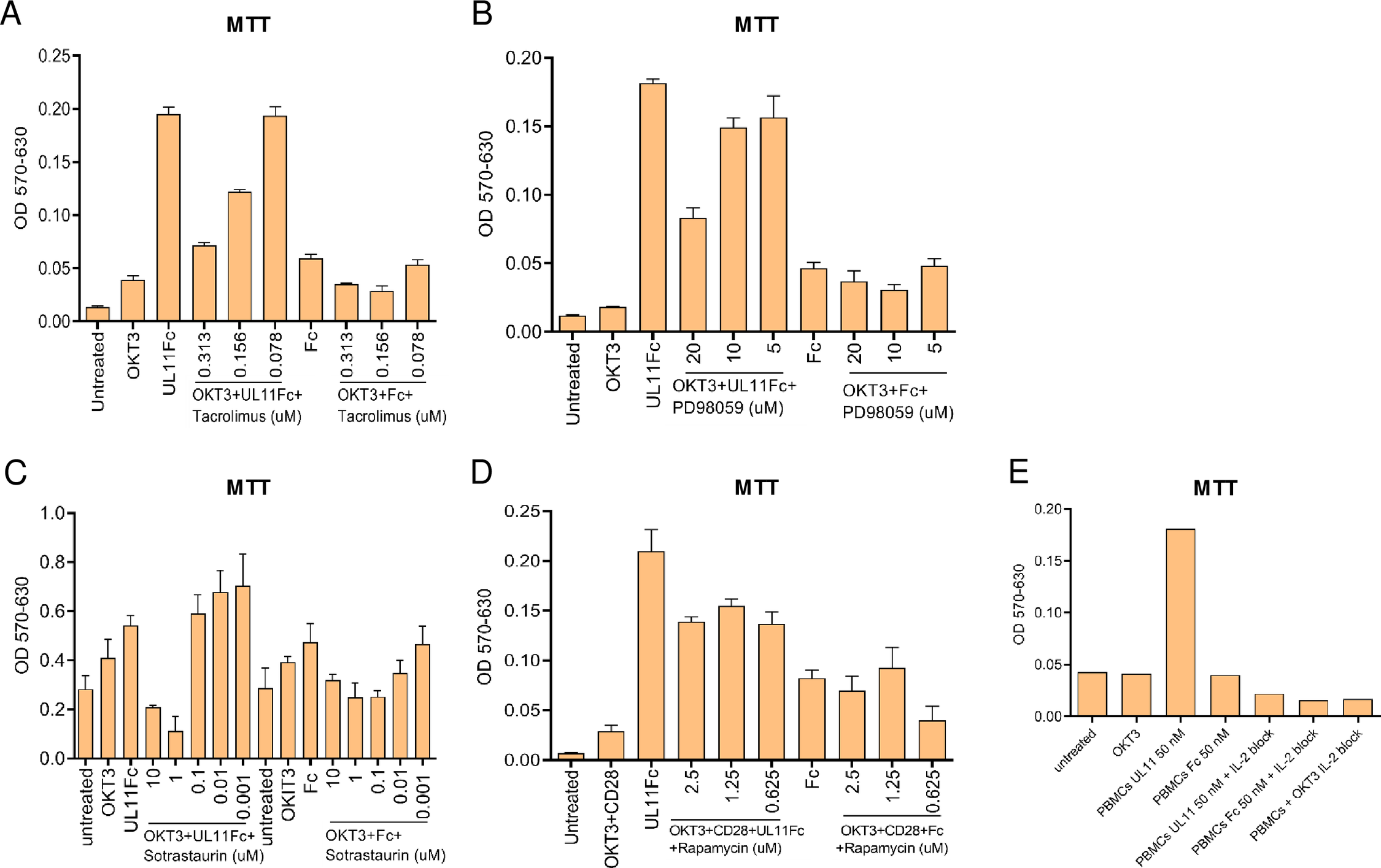

Supplement: FIG S8 [file mbio.02946-22-s0008.tif]

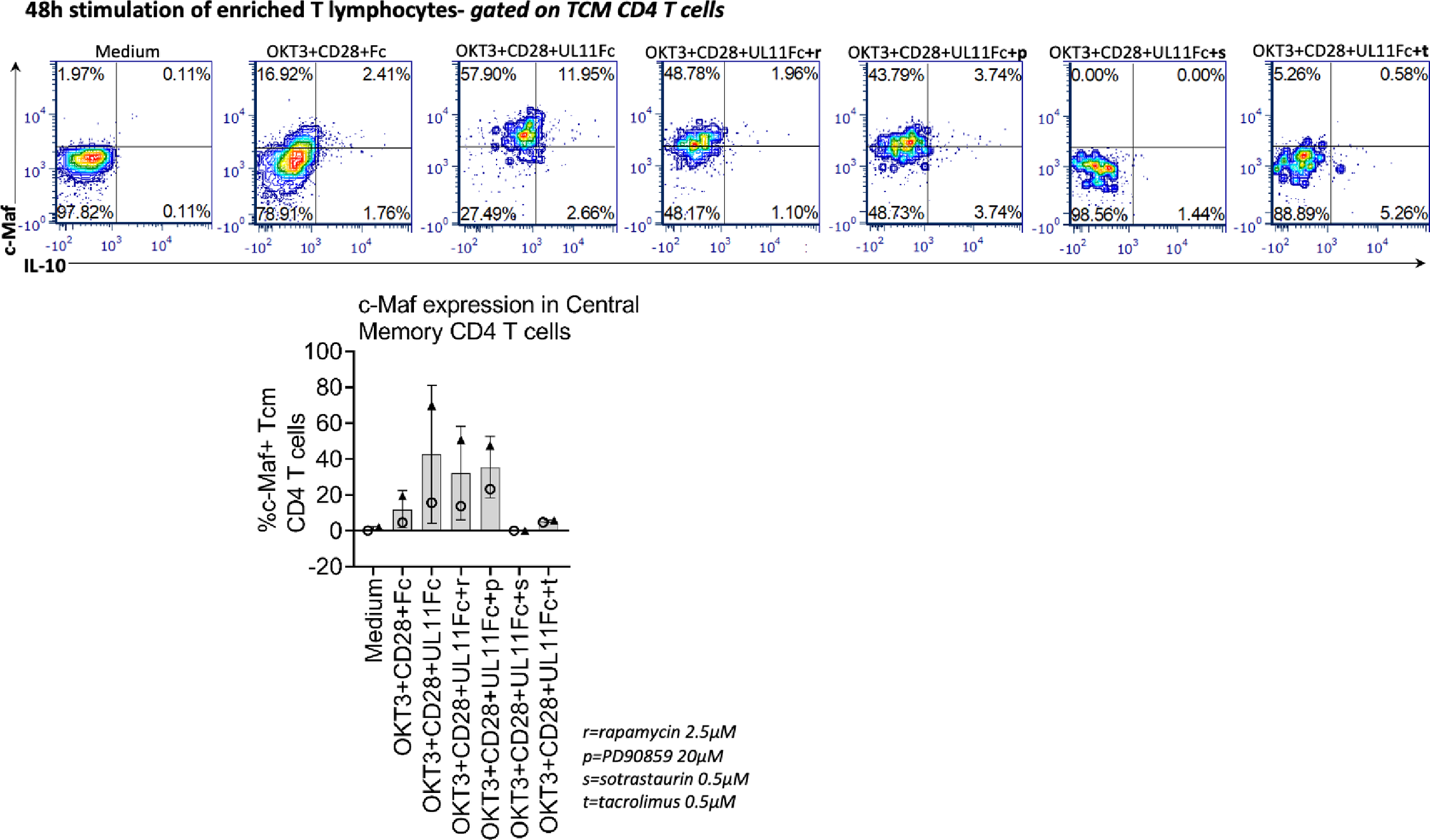

Supplement: FIG S9 [file mbio.02946-22-s0009.tif]
